# Alpha and Omega Classification of β-Lactamase/Transpeptidase-like Superfamily Proteins Based on the Comparison of Their Structural Catalytic Cores

**DOI:** 10.3390/molecules30092019

**Published:** 2025-04-30

**Authors:** Alexander I. Denesyuk, Konstantin Denessiouk, Mark S. Johnson, Vladimir N. Uversky

**Affiliations:** 1 Structural Bioinformatics Laboratory, Biochemistry, InFLAMES Research Flagship Center, Faculty of Science and Engineering, Åbo Akademi University, 20520 Turku, Finland; kdenessi@abo.fi (K.D.); mark.s.johnson@abo.fi (M.S.J.); 2 Department of Molecular Medicine and USF Health Byrd Alzheimer’s Research Institute, Morsani College of Medicine, University of South Florida, Tampa, FL 33612, USA

**Keywords:** β-lactamase, DD-carboxypeptidase, glutaminase, 3D structure, catalytic core, classification

## Abstract

β-Lactamase/transpeptidase-like superfamily proteins are serine proteases that use the Ser–Lys catalytic dyad to carry out their biological functions. Here, we investigate the three known families of β-lactamase/transpeptidase-like superfamily proteins, β-lactamase/D-Ala carboxypeptidase, glutaminase, and Dac-like, and describe the structural catalytic cores that govern the catalytic residues in these proteins. We show that the structural catalytic core of these proteins is a combination of three zones, the mutual three-dimensional arrangement of which correspondingly determines their belonging to one of seven and twenty-four established groups and subgroups.

## 1. Introduction

Serine peptidases/hydrolases are found in all living organisms. These diverse enzymes have been the subject of a significant number of structural studies for many years, reflected in the creation of the MEROPS and ESTHER databases [1,2]. In most cases, these enzymes carry out catalytic activity through the “classical” catalytic triad: Ser(nucleophile)–His(base)–Asp(acid) [3,4].

The sheer number of different three-dimensional (3D) structures of serine peptidases/hydrolases that have been submitted to the Protein Data Bank (PDB) [5] necessitated the search for common structural elements that could be used as a basis to cluster and further classify many unrelated structures that belong to the same structural superfamilies. For example, one common structural element is the Structural Catalytic Core (SCC) around the Ser–His–Asp catalytic triad, which has been used to compare representative 3D structures from trypsin-like serine proteases, α/β-hydrolases, and SGNH hydrolase-like and subtilisin-like superfamily proteins [6,7,8,9]. The functional idea behind the definition of the SCC was that it consisted of a collection of small closed substructures, called “zones”, where each zone (1) included short segments that were repeatedly found in groups of different enzymes, (2) incorporated “key” residues that fulfilled equivalent functional roles, and (3) were interconnected by various atomic interactions that were also similar within those groups of proteins.

In our previous investigation of the SCC, there remained one uncharacterized superfamily of serine proteases with the key representative enzyme of D-alanyl-D-alanine carboxypeptidase that was purposely excluded. This enzyme is an unconventional serine protease in which the classical Ser–His–Asp triad of the active site instead contains either a Ser–Lys or a Ser–Tyr catalytic dyad. Indeed, two separate 3D structures of this protein, PDB ID: 3PTE [10] and PDB ID: 1MPL [11], were used in two separate studies of enzyme function, where, in one study, the role of the general base was shown to be played by Lys [12], and, in the other, by Tyr [13].

According to the Structural Classification of Proteins (SCOP) database [14], D-alanyl-D-alanine carboxypeptidase belongs to the β-lactamase/D-Ala carboxypeptidase structural family and the β-lactamase/transpeptidase-like structural superfamily. Three small characteristic motifs, the Ser-Xaa-Xaa-Lys motif, which is also known as the “S-X-X-K” motif, where Xaa and X signify any amino acid, the (Ser/Tyr)-Xaa-(Asn/Cys) motif, which is also known as the “S-X-N” motif, and the (Lys/His)-(Thr/Ser)-Gly motif, which is also known as the “K-T/S-G” motif, and an additional structural element called the omega(Ω)-loop [15] are present at the active site of all β-lactamase/D-Ala carboxypeptidase family enzymes [16,17,18,19]. The role of the Ω-loop in the catalytic activity of different β-lactamases has been studied in detail [20,21,22].

Due to the unconventional composition and variation of the catalytic triad in D-alanyl-D-alanine carboxypeptidase and the presence of the unique Ω-loop involved in the formation of the active site, the SCC of the β-lactamase/transpeptidase-like superfamily enzymes had been excluded from our previous studies, which we now address here.

In summary, members of the β-lactamase/D-Ala carboxypeptidase family have three standalone structural elements of their catalytic machinery. One, there is a catalytic dyad (Ser–Lys/Ser–Tyr described above) [12,13]. Two, there is a set of atoms of variable residues called the “oxyanion hole”, which is commonly found in serine peptidases [6,7,8,9,12,13]. Three, there is the unique Ω-loop, which is found in the β-lactamase/D-Ala carboxypeptidase family [16,17,18,19]. Following the SCC approach, these three structural elements of catalytic machinery could be incorporated into their respective structural zones, and, if so, the zones could be used as a tool to compare active sites of functionally unrelated “conventional” and “unconventional” triad-type and dyad-type enzymes within the same fold [9].

## 2. Results and Discussion

As described above, the β-lactamase/D-Ala carboxypeptidase family proteins have three standalone structural elements of the catalytic machinery: (1) the catalytic dyad; (2) the oxyanion hole; and (3) the Ω-loop. We also know that these enzymes have three known sequence motifs, Ser-Xaa-Xaa-Lys (or S-X-X-K), (Ser/Tyr)-Xaa-(Asn/Cys) (or S-X-N), and (Lys/His)-(Thr/Ser)-Gly (or K-T/S-G). These data will be taken as the basis for the identification of additional β-lactamase catalytic residue SCC zones. Furthermore, by comparing the respective SCCs in different β-lactamase/transpeptidase-like superfamily proteins, we will group them based on the differences in interactions between key catalytic amino acids and the other structural elements.

### 2.1. Creating a Dataset of the β-Lactamase/Transpeptidase-like Representative Structures

In the SCOP database, the β-lactamase/transpeptidase-like superfamily incorporates three families: (1) the β-lactamase/D-Ala carboxypeptidase family (201 different proteins), (2) the glutaminase family (five different proteins), and (3) the Dac-like family (five different proteins) [14]. For each of these 211 different proteins, one representative PDB 3D structure with the best resolution was selected, thus making a set of 211 representative 3D structures.

### 2.2. SCC Identification with the Example of β-Lactamase CTX-M-14

#### 2.2.1. Conserved Local Motifs at the Basis of the SCC Identification

**Figure 1:**
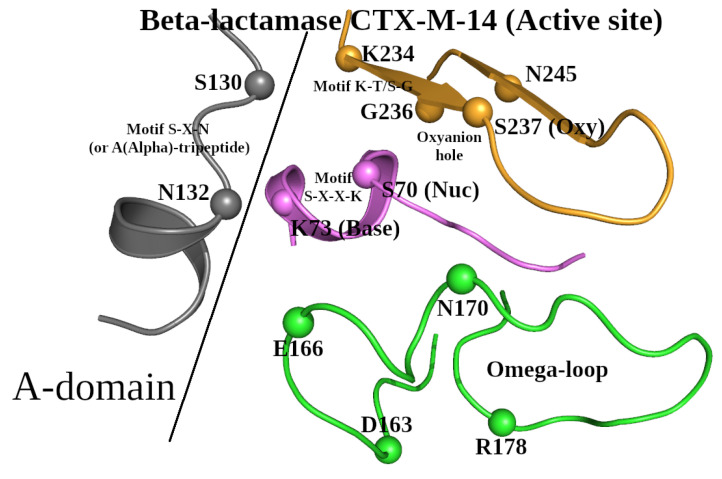
The 3D structure of the active site in β-lactamase CTX-M-14. Three short amino acid segments, Ser_70_-Xaa-Xaa-Lys_73_ (violet), Ser_130_-Xaa-Asn_132_ (grey), and Lys_234_-Thr/Ser-Gly_236_ (orange), show the location of the corresponding sequence motifs. Amino acid names are given as single-letter designations. The letter “X” denotes the possibility of the presence of any amino acid at a given position of the motif. The designation “Oxyanion hole” shows the location of the corresponding functional site formed by atoms N/Ser_70_ and N/Ser_237_. The Omega-loop is shown in green, with the initial (Asp_163_) and final (Arg_178_) amino acids and two functionally important residues (Glu_166_ and Asn_170_) indicated.

Based on the selected representative structure of the β-lactamase/D-Ala carboxypeptidase protein family, we will describe all known conserved structural elements, around which the SCC will be built. Taking into consideration all the structural criteria for creating the dataset of representative structures described above, the 3D structure of β-lactamase CTX-M-14 (PDB ID: 4UA6; 0.79 Å resolution) [23] can be selected to represent the β-lactamase/transpeptidase-like superfamily. This enzyme is a hydrolase and belongs to the class A β-lactamases (http://bldb.eu/S-BLDB.php; accessed on 10 October 2024 [24,25,26]). Typical for all β-lactamases of this class, the active site of CTX-M-14 contains the following characteristic motifs: (1) the S-X-X-K motif is Ser_70_-Xaa-Xaa-Lys_73_; (2) the S-X-N motif is Ser_130_-Xaa-Asn_132_; and (3) the K-T/S-G motif is Lys_234_-Thr/Ser-Gly_236_, and the Ω-loop is Asp_163_-Arg_178_ (Figure 1) [17,20].

For comparison purposes, we will use the sequence and structure of β-lactamase CTX-M-14 as the reference. In the Ser_70_-Xaa-Xaa-Lys_73_ motif, Ser_70_ and Lys_73_ are the catalytic nucleophile (Nuc) and general base (Base), respectively (Figure 1). Together with Lys_73_, Glu_166_ located in the Ω-loop has also been proposed as a potential general base (Figure 1). The amino acids Ser_70_, Lys_73_, Ser_130_, Glu_166_, and Asn_170_ are involved in the enzymatic catalytic activity (Figure 1) [27]. Hydrogen bonds between Ser_130_ and side-chain groups of Ser_70_ and Lys_234_ were also found to be important for protein function [28]. The mutation of Asn_132_ to alanine showed the involvement of this residue in the functional transition-state stabilization [29]. By analogy with the Ω-loop, we will refer to the Ser_130_-Xaa-Asn_132_ motif (the S-X-N motif in Figure 1) located in the A-domain of β-lactamase [30] as the A(Alpha)-tripeptide (Figure 1). Finally, the oxyanion hole is formed by the main-chain nitrogen atoms of two amino acids: the catalytic nucleophile, Ser_70_, and the “Oxy” residue, Ser_237_, which directly follows Lys_234_-Thr-Gly_236_ [30].

#### 2.2.2. The “NucBase-Oxy” Zone

**Figure 2:**
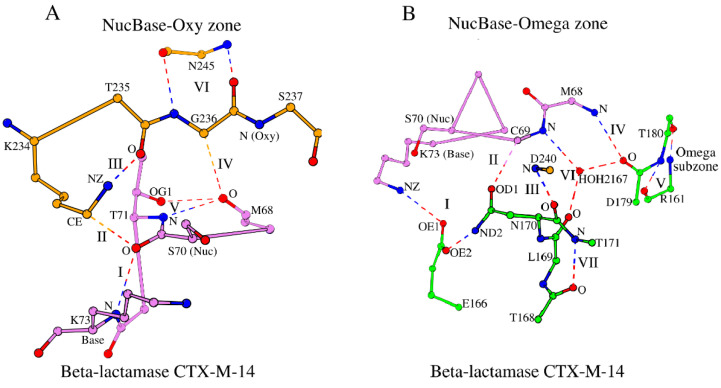
Formation of NucBase-Oxy (**A**) and NucBase-Omega (**B**) zones by peptide segments Met_68_-Lys_73_ (violet), Lys_234_-Ser_237_ (orange), and residues Glu_166_, Leu_169_-Asn_170_, and Asp_179_ of the Ω-subzone (green) in β-lactamase CTX-M-14. The terms “Nuc”, “Base”, and “Oxy” are used to denote the functional characteristics of the catalytic nucleophile Ser_70_, the base Lys_73_, and the atom N/Ser_237_, respectively, involved in formation of the oxyanion hole. The dashed lines show the canonic and weak hydrogen bonds of two types (zone-forming and internally stabilizing). Water HOH_2167_ is shown to stabilize the conformation of the NucBase-Omega zone.

Let us consider interactions between the local substructures that govern the key motifs (Ser_70_-Xaa-Xaa-Lys_73_ (the S-X-X-K motif), Ser_130_-Xaa-Asn_132_ (the S-X-N motif), and Lys_234_-Thr/Ser-Gly_236_ (the K-T/S-G motif)) and the Ω-loop described above. The hexapeptide Met_68_-Lys_73_, which incorporates the catalytic nucleophile Ser_70_ and the catalytic base Lys_73_, is a unique structural segment that not only contains a catalytic dyad but also forms two separate local mini-networks of hydrogen bonds and weak interactions with the oxyanion hole and the Ω-loop, respectively. We will designate the Met_68_-Lys_73_ hexapeptide as the “NucBase” hexapeptide. The NucBase hexapeptide and the Lys_234_-Ser_237_ tetrapeptide, which incorporates the Oxy residue Ser_237_, form a local interconnected substructure that incorporates the catalytic residues and the oxyanion hole and is internally bound by a mini-network of hydrogen bonds and weak interactions (Table 1 and Table S1). We will refer to this standalone substructure as the “NucBase-Oxy zone” (Figure 2A). The contacts marked as I (between Ser_70_ and Lys_73_), III (between Lys_234_ and Thr_235_), and V (between Met_68_ and Thr_71_; all defined in Table S1 and shown in Figure 2A) are the canonical hydrogen bonds, while the contacts marked as II (between Ser_70_ and Lys_234_) and IV (between Met_68_ and Gly_236_) are a weak hydrogen bond (Derewenda [31] has written a comprehensive review on weak hydrogen bonds in the 3D structures of proteins and nucleic acids) and a van der Waals interaction, respectively. In Table S1, it is easy to separate standard hydrogen bonds from weak ones. The cut-off distance for a canonical hydrogen bond is ≤3.3 Å (Table S1, row 1), while for a weak C-H•O hydrogen bond, the cut-off distances are usually slightly larger (≤4.0 Å) with the C-H•O angle strictly ≥130° [32].

The interaction between O/Met_68_ and CA/Gly_236_ (the interaction marked as IV in Table S1 and Figure 2A) locks the ends of the “circular” structure of the NucBase-Oxy zone and also affects the relative arrangement of the functionally important nodes of the oxyanion hole, N/Ser_70_ and N/Ser_237_, which should keep their position intact during catalysis (Figure 2A). Indeed, according to the PDBsum database [33] and the Ligplot^+^ v.2.2 tool [34], in the ligand-bound structures of β-lactamase CTX-M-14 (PDB ID: 4UA9) the two atoms of the oxyanion hole do interact with the ligand, and neither their position nor the interaction between O/Met_68_ and CA/Gly_236_ (interaction IV in Table S1) changes when comparing the ligand-free (PDB ID: 4UA6) and ligand-bound (PDB ID: 4UA9) forms of the enzyme. Thus, the observed distance between O/Met_68_ and CA/Gly_236_ does show local structural conservation of the NucBase-Oxy zone conformation but also reflects the requirements of protein function.

The rigid main-chain-based dual interaction between Gly_236_ and Asn_245_ (N/Gly_236_-O/Asn_245_ and O/Gly_236_-N/Asn_245_; interaction VI in Table S1 and Figure 2A) is the only dual-bond interaction that the residues of the NucBase-Oxy zone form with the rest of the protein and not among residues of the zone or the protein ligand (residue Asn_245_ is not part of the NucBase-Oxy zone). However, Gly_236_ and Asn_245_ are located on two adjacent antiparallel β-strands of the β-sheet, of which one β-strand containing the residue Gly_236_ is positioned on the edge of the β-sheet (Figure 1), thus linking the NucBase-Oxy zone to the overall protein fold. The Gly_236_–Asn_245_ dual-bond interaction is typical of rigid secondary structures, such as β-sheets. As a result, the positions of the functionally important atoms of N/Ser_237_ and O/Thr_235_ (the Oxy atom and interaction III in β-lactamase CTX-M-14, respectively) are stabilized by this dual-bond interaction VI (Figure 2A).

#### 2.2.3. The NucBase-Omega Zone and Its Omega (Ω) Subzone

In β-lactamase CTX-M-14, the NucBase hexapeptide Met_68_-Lys_73_ and three segments of the Ω-loop, Glu_166_, Leu_169_-Asn_170_, and Asp_179_, form the NucBase-Omega zone (Figure 2B; Table 1 and Table S2). Similar to the NucBase-Oxy zone, the NucBase-Omega zone is a standalone structure—an internally interlocked conserved substructure—that creates and supports the connection between the catalytic residues and the Ω-loop. Thus, while the NucBase-Oxy zone extends from the catalytic dyad to the oxyanion hole, the NucBase-Omega zone extends to the Ω-loop. The NucBase-Omega zone also contains a water molecule “mediator” (HOH_2167_ in Figure 2B), which forms three hydrogen bonds with the constituent structural elements of the zone, one hydrogen bond per element (Table S2, column V). Figure 2B shows all interactions that interlock the NucBase-Omega zone. These interactions are designated by numbers I through VII and are repeatedly found in proteins of the β-lactamase/transpeptidase-like superfamily (Table S2). In the NucBase-Omega zone, contacts I, IV, and VI are typically canonical hydrogen bonds, contact II is a weak hydrogen bond, and contact V consists of two strong hydrogen bonds connecting two key amino acids and in the majority of cases is an antiparallel main-chain O-N + N-O link (Table S2).

Our knowledge of the NucBase-Omega zone allowed us to separately pinpoint the Ω-subzone, the “pocket” of the NucBase-Omega zone, which incorporates the Ω-loop. This zone-based structural approach further allowed us to identify scaffolds supporting the Ω-loop and the other key functional elements without regard to their sequence length. In the β-lactamase CTX-M-14, however, the Arg_161_-Thr_180_ localization of the Ω-subzone does not differ much from the Asp_163_-Arg_178_ localization of the Ω-loop [29], but this is not always the case.

#### 2.2.4. SCC as a Structural Association of the NucBase-Oxy and NucBase-Omega Zones and the A-tripeptide Link

**Figure 3:**
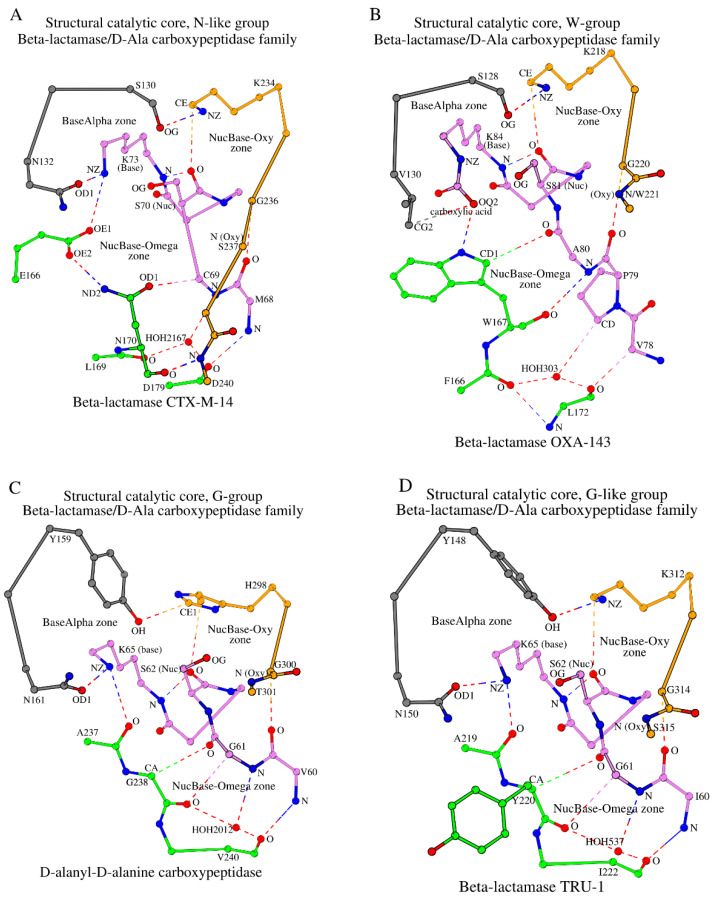
Formation of the SCCs by combining three zones, NucBase-Oxy (orange and violet), NucBase-Omega (green and violet), and BaseAlpha (grey and violet), in the four most numerous groups (N-like (**A**), W (**B**), G (**C**), and G-like (**D**)) of the β-lactamase/D-ala carboxypeptidase family. The dashed lines show the canonic and weak hydrogen bonds of two types (zone-forming and internally stabilizing). Conserved water molecules are shown to stabilize the conformation of the NucBase-Omega zones.

The combination of bound NucBase-Oxy and NucBase-Omega zones of β-lactamase CTX-M-14 is shown in Figure 3A. The amino acid chain direction of the NucBase segment Met_68_-Lys_73_ is antiparallel to the direction of the Oxy segment Lys_234_-Asp_240_ and the Ω-subzone, whose chain directions coincide.

Moreover, at one end of the arrangement, shown at the bottom of Figure 3A, the NucBase segment, Oxy segment, and Ω-subzone structurally converge, while at the opposite end they diverge (Figure 3A). Structurally, the divergent part of the NucBase-Omega-Oxy arrangement (upper left part of Figure 3A) is connected by the A-tripeptide Ser_130_-Asn_132_. As was shown in Figure 1, the A(Alpha)-tripeptide in β-lactamase CTX-M-14 is the S-X-N motif, but in the other representative structures of the same superfamily its amino acid composition can vary (shown as “Alpha” in Table 1).

**Table 1:** Structural Catalytic Core (SCC) in 25 β-lactamase/transpeptidase-like superfamily representative proteins.

N	PDB ID	R (Å)	Protein	NucBase	Alpha	Omega	Oxy	Mediator	Ref.
Superfamily: β-lactamase/transpeptidase-like									
Family: β-lactamase/D-Ala carboxypeptidase									
N-like group (Class A) (79)									
SNN subgroup (67)									
1	4UA6_A	0.79	β-lactamase CTX-M-14	68 MCSTSK 73	130 SDN 132	E166, 169 LN••D 179	234 KTGSGD 240	HOH2167	[23]
SNS subgroup (5)									
2	5F82_A	0.96	Carbapenemase GES-5	62 MGSTFK 67	125 SDN 127	E161, 164 MS••D 174	229 KTGTCA 234	HOH498	[35]
SNG subgroup (2)									
3	2QPN_A	1.10	Carbapenemase GES-1	62 MCSTFK 67	125 SDN 127	E161, 164 MG••D 174	229 KTGTCA 234	HOH338	[36]
SSN subgroup (2)									
4	7DDM_A	1.20	β-lactamase PenA39	68 FCSTFK 73	130 SDS 132	E166, 169 LN••D 179	234 KTGTGD 240	HOH476	[37]
SGN subgroup (3)									
5	5NJ2_A	1.19	β-lactamase BlaC	68 FCSTFK 73	128 SDG 130	E168, 171 LN••D 181	236 KTGTGD 242	HOH547	[38]
W-group (Class D) (45)									
SVW subgroup (36)									
6	5IY2_B	1.15	β-lactamase OXA-143	78 VPASTFK 84	128 SAV 130	166 FW••L 172	218 KSGW 221	HOH303	[39]
SIW subgroup (5)									
7	6W5E_A	1.30	β-lactamase BSU-2	98 TPQSTFK 104	149 SAI 151	187 FW••L 193	239 KTGT 242	HOH450	[40]
SLW subgroup (4)									
8	6N1N_A	1.60	β-lactamase STD-1	62 LPASTFK 68	113 SAL 115	151 FW••L 157	203 KTGW 206	HOH510	[41]
W-group (5)									
SNW subgroup (4)									
9	2IWB_A	1.80	Methicillin resistance mecR1 protein	388 SPNSTYK 394	439 SVN 441	476 YW••L 482	528 KTGT 531	HOH2115	[42]
STW subgroup (1)									
10	1NRF_A	2.50	Regulatory protein BlaR1	399 APASTYK 405	450 STT 452	487 YW••L 493	539 KTGT542	HOH738	[43]
G-group (23)									
YNG subgroup (4)									
11	1YQS_A	1.05	D-alanyl-D-alanine carboxypeptidase	60 VGSVTK 65	159 YSN 161	237 AG••V 240	298 HTGT 301	HOH2012	[44]
SNG subgroup (17)									
12	5ZQA_A	1.55	Lmo2812 protein	56 IASLSK 61	118 SAN 120	158 SG••A 167	222 KTGF 225	HOH515 HOH418	[45]
SCG subgroup (1)									
13	1ES5_A	1.40	DD-transpeptidase	33 TGSTTK 38	96 SGC 98	143 DG••N 150	213 KTGA 216	HOH479 HOH347	[46]
YSG subgroup (1)									
14	1WYB_A	1.80	6-aminohexanoate-dimer hydrolase	110 LMSVSK 115	215 YCS 217	266 HG••V 269	342 GIGI 345	CG/L109 CD1/L109	[47]
G-like group (39)									
YNY subgroup (Class C) (33)									
15	6FM6_A	1.05	β-lactamase TRU-1	60 IGSVSK 65	148 YSN 150	219 AY••I 222	312 KTGS 315	HOH537	[48]
YNA subgroup (1)									
16	1EI5_A	1.90	D-aminopeptidase	60 ICSVSK 65	153 YCN 153	225 DA••I 228	287 HGGA 290	HOH531	[49]
YLA subgroup (2)									
17	1CI9_A	1.80	Esterase EstB	73 LASVTK 78	181 YSL 183	274 GA••M 277	348 WGGV 351	HOH1050	[50]
YHQ subgroup (1)									
18	4IVK_A	1.80	Carboxylesterase	98 IYSMSK 103	218 YGH 220	295 GQ••M 298	381 WGGA 384	HOH666	[51]
YPH subgroup (1)									
19	6KJC_A	2.30	Lovastatin esterase	55 LASATK 60	170 YGP 172	252 GH••L 255	344 WGGG 347	Y54	[52]
SNM subgroup (1)									
20	2BG1_A	1.90	Penicillin-binding protein 1b	457 SPASTTK 463	516 SWN 518	555 PM••I 560	651 KTGT 654	OG/S457	[53]
Q-like group (Class A) (6)									
SNQ subgroup (5)									
21	6V4W_A	1.29	β-lactamase CPA-1	66 MQSVFK 71	131 SDN 133	E167, 177 –Q••N 180	235 KTGS 238	HOH498	[54]
SNT subgroup (1)									
22	5TFQ_A	1.07	β-lactamase HGB-2	46 LLSVFK 51	112 SDN 114	E148, 157 –T••N 160	215 KTGS 218	HOH472	[55]
Inactive β-lactamase group (2)									
GKN subgroup (2)									
23	5IHV_A	1.10	β-lactamase *B. ambifaria* MC40-6	45 LCGTYA 50	107 GDK 109	E143, 146 LN••D 156	211 KAGTGG 216	HOH484	[56]
Family: Glutaminase									
C-group (5)									
ONC subgroup (5)									
24	1U60_A	1.61	Glutaminase 1	64 LESISK 69	115 LVN 117	E161, Y192, 196 –C••T 198	259 KSGV 262	N/A	[57]
Family: Dac-like									
G(Dac-like)-group (5)									
SNG subgroup (5)									
25	2EX2_A	1.55	D-alanyl-D-alanine carboxypeptidase DacB	59 LPASTQK 65	306 SDN 308	357 SG••N 363	417 KTGS 420	HOH1005	[58]

N/A, Not Available. The symbol “••”, replacing one or more residues within Ω-subzones, is used for the convenient construction of their structural alignment. The symbol “–” indicates that three proteins lack a residue in the corresponding Ω-subzone that is involved in the formation of the SCC.

Nevertheless, we will show that the structural role of the A-tripeptide remains the same. In the β-lactamase CTX-M-14, two side-chain oxygen atoms of the terminal residues of the A-tripeptide form hydrogen bonds with the NZ atoms of the Lys_73_ (catalytic base) and Lys_234_ (Table S2, interaction VIII), and, thus, the A-tripeptide forms a conserved link, the A-tripeptide link, between these two positions. Taken together, the combination of the NucBase-Oxy zone, NucBase-Omega zone, and A-tripeptide link form the Structural Catalytic Core (SCC) of β-lactamase CTX-M-14 and the rest of the β-lactamase/D-Ala carboxypeptidase family. It consists of 19 amino acids and a water molecule mediator, which is incorporated into the NucBase-Omega zone (Table 1; Figure 3A).

### 2.3. The SCC in Proteins of the β-Lactamase/D-Ala Carboxypeptidase Family: Groups, Subgroups, and Classes

After examining the β-lactamase CTX-M-14, the remaining 200 representative 3D structures from the β-lactamase/D-Ala carboxypeptidase family of the β-lactamase/transpeptidase-like superfamily proteins were similarly analyzed for the SCCs formed by four main structural elements and incorporating seven key functional amino acids. The results are summarized in Table 1, Tables S1 and S2.

All representative structures in Table 1, Tables S1 and S2 structurally aligned with respect to the position of equivalent key functional amino acids and equivalent amino acid segments in the protein structure. Unlike a standard sequence alignment, a structural alignment shows the alignment of equivalent positions within the protein structure, which may or may not contain similar amino acids. Subsequent identification of similar amino acids in the key positions of a structural alignment can identify key amino acids that can serve as a good basis for structure classification. We have analyzed the structural alignments in Table 1, Tables S1 and S2 found three such key structural positions that can serve as the basis for classifying and naming SCC groups in all proteins of the β-lactamase/transpeptidase-like superfamily. Consequently, 199 representative structures of this family were divided into six groups and 23 subgroups as described below.

The three key structural positions that were chosen for the naming of groups of the SCC are those equivalent to β-lactamase CTX-M-14 positions 130 (Ser_130_ in CTX-M-14), 132 (Asn_132_ in CTX-M-14), and 170 (Asn_170_ in CTX-M-14). Positions 130 and 132 represent the beginning and the end of the A-tripeptide link, respectively, while position 170 is the key interacting amino acid from the Ω-loop (Figure 1; Table 1 and Tables S1). In two representative structures, it was not possible to fully identify some of these positions. In β-lactamase SPH-1 (PDB ID: 4EWF), it was not possible to identify a residue of the Ω-loop located at a position equivalent to position 170 of CTX-M-14. In penicillin binding protein 1A from *S. Pneumoniae* (PDB ID: 2V2F), it was not possible to determine the beginning and end of the Ω-subzone according to our accepted procedure (Section 2.2.3).

#### 2.3.1. The N-like Group and Its Subgroups

According to the three amino acids described above, the β-lactamase CTX-M-14 is a representative member of the “SNN” subgroup (S_130_-N_132_-N_170_). In addition to the structural and functional properties described in Section 2.2.1, Ser_130_ and Asn_132_ are directly involved in ligand binding [33,34]. The conserved asparagine at position 170, Asn_170_, is one of the most important residues involved in the interaction of the Ω-subzone with both the Met_68_-Cys_69_ dipeptide and the Ser_237_-Asp_240_ tripeptide.

Together with the β-lactamase CTX-M-14, there are 67 representative 3D structures of β-lactamase/D-Ala carboxypeptidase family proteins that belong to the SNN subgroup (shown in round brackets in Table 1; also shown in column “Sum” in Table S1). All 67 proteins have almost identical SCCs and seven key functional amino acids are identical at positions 70, 73, 130, 132, 166, 170, and 234 (Figure 1). Additionally, structurally analogous SCCs are observed in four more subgroups (SSN, SGN, SNS, and SNG), which have undergone single amino acid mutations at position 132 or 170 to serine or glycine, but otherwise are the same as the SNN subgroup (Table 1 and Tables S1). These five subgroups together contain 79 representative structures that belong to class A β-lactamases and together constitute the “N-like” group. The “N-like” name comes from the fact that, even though residues at positions 132 and 170 can be mutated to serine or glycine in some subgroups, the structure of the SCC in all members of this group remains similar to SNN.

Finally, some proteins of the N-like group contain a disulfide bond between the cysteine preceding the nucleophile position and the central cysteine of the Ser_237_-Cys_238_-Asp_240_ tripeptide. For example, such a disulfide bond is observed in the carbapenemase GES-1 (Table 1, row 3).

#### 2.3.2. The W-Group

The second, the “W-group” (class D β-lactamases (http://bldb.eu/S-BLDB.php; accessed on 10 October 2024 [24,25,26])), included 45 structures where tryptophan occupies position 170 (position numbering according to CTX-M-14; see the “Omega” column in Table 1) instead of asparagine. This group can be divided into three subgroups (SVW, SIW, and SLW), where, according to our accepted naming, position 130 is occupied by serine (S), position 132 is occupied by valine (V), isoleucine (I), or leucine (L), and position 170 is occupied by tryptophan (W). The three subgroups consist of 36, 5, and 4 representative structures, respectively (column “Sum” in Table S1). The appearance of branched hydrophobic residues in the W-group instead of a small or polar residue as in the N-like group is related to the protein function of these enzymes. For example, in β-lactamase OXA-24/40 (PDB ID: 5TG4), the appearance of valine at position 130 (132 in CTX-M-14) is consistent with the need to contact the hydrophobic aromatic ring of the ligand [33,34]. In addition to the 45 structures mentioned above, the W-group also includes 5 additional representative structures within two subgroups, SNW (e.g., methicillin resistance mecR1 protein) and STW (e.g., regulatory protein BlaR1), that are not β-lactamases and, thus, are not included in the β-Lactamase database (http://bldb.eu/S-BLDB.php; accessed on 10 October 2024). Here, again, position 132 is occupied by a small polar amino acid.

The structure of β-lactamase OXA-143 (PDB ID: 5IY2) can be taken as the representative for the W-group because the crystal structure has the highest resolution (1.15 Å) [39]. Comparing SCCs between CTX-M-14 and OXA-143, we were able to draw conclusions about observed differences in the SCCs between the N-like group and the W-group. The most noticeable differences in their organization of the SCCs are linked to the construction of their NucBase-Omega zones (Figure 3B vs. Figure 3A). The tryptophan side-chain group (shown in green in Figure 3B) is significantly larger in size compared with the asparagine side-chain group (shown in green in Figure 3A) and does not contain an oxygen atom. Therefore, instead of the OD1/Asn_170_-CA/Cys_69_ weak hydrogen bond seen in β-lactamase CTX-M-14, the CD1/Trp_167_-O/Ala_80_ contact is present in β-lactamase OXA-143 (column II in Table S2). In addition, a hydrogen bond is formed directly between tryptophan and the catalytic base (NE1/Trp_167_–OQ2/Lys_84_; Table S2, column I). Taken together, the need for an analog of Glu_166_ disappears. The physical “shift” of the hydrogen bond CD1/Trp_167_-O/Ala_80_ toward the catalytic nucleophile in OXA-143 and the rest of the W-group proteins (see CD1/Trp_167_-O/Ala_80_ in OXA-143 in Figure 3B vs. OD1/Asn_170_-CA/Cys_69_ in CTX-M-14 in Figure 3A) is coupled by the appearance of an additional conventional hydrogen bond (O/Trp_167_–N/Ala_80_, OXA-143; Table S2, column III). It is important to note that, while in the β-lactamase CTX-M-14 the main-chain oxygen of Asn_170_ forms a hydrogen bond with N/Asp_240_ of the Oxy segment, in the β-lactamase OXA-143 the existence of O/Trp_167_–N/Ala_80_ eliminates the possibility of contacts between tryptophan and the last two residues of the Oxy segment and effectively removes those residues from the SCC (Table 1). As we will show below, interactions of the amino acid at position 170 (Trp in the W-group) with the residue preceding the catalytic nucleophile, together with the “shortening” of the Oxy segment in the SCC, are specific structural markers of the β-lactamase/transpeptidase-like superfamily proteins.

Another important difference between the SCCs of the two groups of β-lactamases is the replacement of asparagine (Asn_132_ in CTX-M-14 from the N-like group) in the A-tripeptide with valine (Val_130_ in OXA-143 from the W-group) (Figure 3B vs. Figure 3A). However, even in the W-group, the A-tripeptide link is formed due to the contact of valine with carboxylic acid, which is covalently linked to the catalytic Lys_84_ (Figure 3B). Note that, in four proteins out of the five belonging to the W-group β-lactamases, asparagine is located instead of valine in the A-tripeptide, and the formation of the BaseAlpha zone occurs in the same way as in class A β-lactamases (Table S2, column VIII). Finally, the SCC of the W-group proteins consists of 17 amino acids instead of the 19 seen in the N-like group (class A of the β-lactamases) (Table 1).

In Section 2.3.1, it was shown that, in some N-like group proteins, there is a disulfide bond that stabilizes the conformation of the NucBase-Oxy zone. In particular, the cysteine located before the catalytic nucleophile in the amino acid sequence (position “nuc-1”) takes part in its formation. Thus, the amino acid at position nuc-1 is one of the key residues in the formation of both the NucBase-Oxy zone and the NucBase-Omega zone and, therefore, the entire SCC. Further evidence for the important structural role of the nuc-1 residue in the formation of the NucBase-Omega zone is seen in W-group proteins. In β-lactamase BSU-2 and methicillin resistance mecR1 protein, instead of the frequently observed alanine, the nuc-1 position is occupied by glutamine or asparagine (Table 1, rows 7 and 9, respectively). In these two proteins, the side-chain group of the nuc-1 residues forms two hydrogen bonds with glutamine/glutamic acid at the C-terminus of the Ω-subzone (Table S2, rows 7 and 9, columns III and V).

#### 2.3.3. The G-Group

The third group of proteins (23 representative structures) has glycine at sequence position 170 (position numbering according to CTX-M-14); thus, it is referred to as the “G-group”. The G-group consists of four subgroups with D-alanyl-D-alanine carboxypeptidase as its representative (PDB ID: 1YQS, R = 1.05 Å) [44]. The SCC of D-alanyl-D-alanine carboxypeptidase is shown in Figure 3C.

Based on the naming introduced here, D-alanyl-D-alanine carboxypeptidase belongs to the YNG subgroup (Table 1). Unlike the N-like group and W-group proteins, D-alanyl-D-alanine carboxypeptidase has two internal weak hydrogen bonds, CA/Gly_238_–O/Gly_61_ and O/Gly_238_–CA/Gly_61_ (in place of the CD1/Trp_167_–O/Ala_80_ and O/Trp_167_–N/Ala_80_ bonds in β-lactamase OXA-143), which together account for approximately 60% of one standard hydrogen bond. A water molecule mediator is also present in the G-group proteins (Figure 3C; Table S2). Due to the absence of a side chain in Gly_238_, the main-chain oxygen of Ala_237_ is involved in the NucBase-Omega zone-forming contacts with the catalytic base (Table S2, column I). The conformation of the side-chain group of the catalytic base is stabilized by its interaction with Asn_161_ of the A-tripeptide via hydrogen bonding (Table S2, column VIII). An additional zone-forming bond O/Val_240_–N/Val_60_ is formed between the terminal amino acids of the NucBase and Ω-subzone segments (Figure 3C; Table S2, column IV). As a result, the lengths of the Oxy segments in carboxypeptidase and β-lactamase OXA-143 coincide. However, the carboxypeptidase Oxy segment has one noticeable difference compared with the respective segments from the N-like and W-groups: the initial amino acid of this segment in the G-group is not necessarily lysine (histidine in D-alanyl-D-alanine carboxypeptidase, Table 1). Otherwise, the NucBase-Oxy zones are very similar in the three groups of proteins (Figure 3A–C; Table S2).

In D-alanyl-D-alanine carboxypeptidase, interactions between the NucBase-Oxy zone and the A-tripeptide link occur through the Tyr_159_–His_298_ pair, which is unique for the G-group of proteins. If we consider all four subgroups of the G-group simultaneously, then in two out of the four subgroups the serine at the first position of the A-tripeptide is replaced by tyrosine (Table 1). The SCC of the G-group consists of 16 amino acids.

Finally, the G-group also contains a representative protein, 6-aminohexanoate-dimer hydrolase, with methionine at the nuc-1 position (Table 1), which interacts with the Oxy residue and, thus, participates in stabilizing the NucBase-Oxy zone.

#### 2.3.4. The G-like Group

The G-like group includes 39 proteins whose SCC is nearly the same as the SCC of the G-group, despite the fact that they do not have glycine at position 170 (see Figure 3D vs. Figure 3C; position numbering according to CTX-M-14; Table 1). Proteins of the G-like group are divided into six subgroups, of which the YNY subgroup (33 representative proteins) is the largest. β-lactamase TRU-1 (PDB ID: 6FM6, 1.05 Å resolution) [48] from the YNY subgroup was selected as the representative structure of the G-like group. Proteins of the YNY subgroup belong to class C β-lactamases (http://bldb.eu/S-BLDB.php; accessed on 10 October 2024 [24,25,26]). The only feature of this group that distinguishes it from the other groups is the presence of tryptophan at the first position of the Oxy segment in some subgroups instead of a lysine (see “Oxy” column in Table 1, lines 17, 18, and 19). In such structures, the NE1 atom of tryptophan substitutes for the NZ atom of lysine in all respective interactions between the A-tripeptide and the Oxy segment (Table S2, column VIII). Tyrosine at the first position of the A-tripeptide is conserved with very few exceptions in the G-like group (see the “Alpha” column in Table 1). Similar to the G-group, the SCC of the G-like group proteins is constructed from 16 amino acids.

#### 2.3.5. The Q-like Group

Unlike the groups described above, the Q-like group is relatively small. It contains six representative structures from two subgroups (Table 1). The SNQ subgroup is dominant. It includes five representative structures. The last representative structure of this group (PDB ID: 5TFQ) has threonine at position 170 (position numbering according to CTX-M-14; Thr_158_ in 5TFQ), and, thus, formally forms the SNT subgroup. Due to the predominance of glutamine at position 170, this group of proteins is referred to as the “Q-like” group.

**Figure 4:**
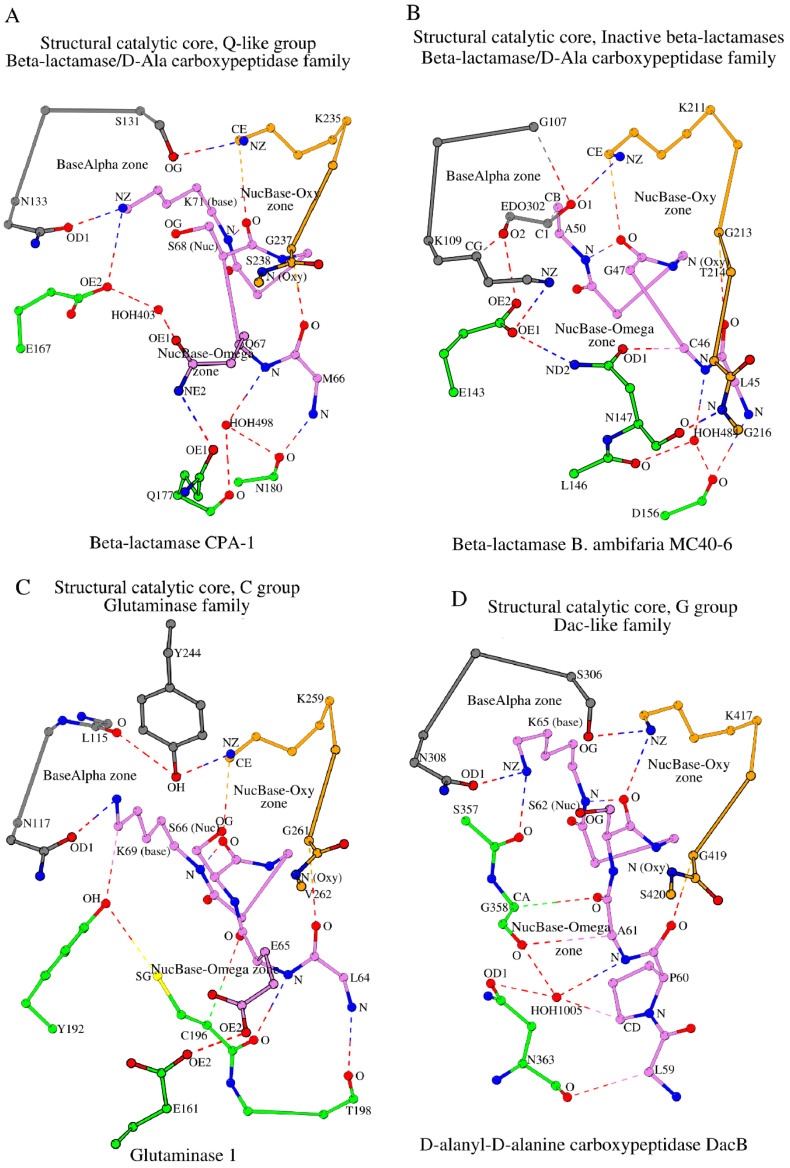
Formation of the SCCs in two minor groups, Q-like (**A**) and inactive β-lactamases (**B**), of the β-lactamase/D-ala carboxypeptidase family as well as in proteins of the glutaminase (**C**) and Dac-like (**D**) families. EDO_302_ is 1,2 ethanediol (**B**).

As with the N-like group, the Q-like group proteins are class A β-lactamases (http://bldb.eu/S-BLDB.php; accessed on 10 October 2024). However, there are several fundamental differences between the SCCs of the N- and Q-like groups. Let us compare the SCCs of β-lactamases CTX-M-14 and CPA-1 (PDB ID: 6V4W, 1.29 Å resolution) [54] (see Figure 4A vs. Figure 3A). Firstly, there is predominantly glutamine at position 170 in the Q-like group that is never found in the N-like group. Secondly, the side-chain group of this glutamine (Gln_177_ in CPA-1) contacts the side-chain group of Gln_67_, adjacent to the catalytic nucleophile Ser_68_. An analogous interaction does not exist in any of the groups discussed above. Gln_67_ and HOH_403_ act as intermediaries in the contact of Gln_177_ with the functionally important Glu_167_, a structural analog of Glu_166_ in the β-lactamase CTX-M-14 (see Section 2.2.1). Finally, unlike the N-like group proteins, the Oxy segment of the Q-like group contains only four residues, similar to some other groups shown in Table 1. The SCC of the Q-like group proteins consists of 16 amino acids in total.

#### 2.3.6. The Group of “Inactive” β-Lactamases, i.e., Those Unable to Perform Catalysis

All proteins we have considered so far are enzymatically active. However, the β-lactamase/D-ala carboxypeptidase family includes two structures whose proteins are inactive due to amino acid changes at the catalytic nucleophile and base positions, and yet they have the same fold and belong to the same protein family (see “Inactive β-lactamase group” in Table 1). Let us consider how changes at the two catalytically important amino acid positions affect the SCC of β-lactamase *B. ambifaria* MC40-6 (PDB ID: 5IHV, 1.10 Å resolution) [56]. Essentially, the SCC in this protein is similar to the SCC of the N-like group, class A β-lactamases (see Figure 4B vs. Figure 3A). Replacing serine with glycine at the catalytic nucleophile position (Gly_47_ in Figure 4B) has no effect on the structure of the SCC because of the main-chain interactions and the small size of glycine. All contacts lost due to the exchange of the catalytic base to alanine (Ala_50_ in Figure 4B) are formed instead by the bound small molecule, ethane 1,2-diol (used in the crystallization solution, EDO_302_), which maintains the local conformation. Finally, substitution of asparagine for lysine at position 132 of the A-tripeptide has very little effect on the conformation of the NucBase-Omega zone and the A-tripeptide link. Like β-lactamase CTX-M-14, the SCC of β-lactamase *B. ambifaria* MC40-6 consists of 19 amino acids.

### 2.4. Some Important Observations

Summarizing the structural comparison of SCCs in proteins of the β-lactamase/D-Ala carboxypeptidase family from the N-like group (Class A), W-group (Class D), G-group, G-like group, Q-like group (Class A), and inactive β-lactamase group (first 23 proteases from Table 1), we can so far conclude:

*(1)*  *Structural Catalytic Cores (SCCs) in N-like and W-groups have key differences*. In the W-group, the appearance of key tryptophan (W) at position 170 instead of asparagine (N) at the equivalent position in the N-like group (see W167 in Figure 3B vs. N170 in Figure 3A), which is also reflected in the naming of the groups, leads to the two main structural changes in the protein SCC between the two groups. Due to the larger side-chain size and additional interactions between tryptophan and the NucBase structural segment (Figure 3B vs. Figure 3A): (a) the intermediate role of E166 and the equivalent residues, which are invariant in the N-like group (Table 1), disappears; but instead (b) the oxyanion (Oxy) segment in the W-group is shorter as it lacks the two last residues and their respective interactions;
*(2)*  *SCCs in G- and G-like groups are closer to the W-group rather than to the N-like group*. In G- and G-like groups, there is no side chain in glycine at the key position 170 (G238 in Figure 3C; position numbering according to 4UA6). As a consequence, in these groups it is not the glycine at position 170 that participates in the NucBase-Omega interactions but the amino acid at position 169 (A237 in Figure 3C), which, similarly to as seen in the W-group, removes the necessity of E166 mediation (Figure 3A) and shortens the oxyanion (Oxy) segment. In this respect, the SCC of G- and G-like groups is structurally closer to the SCC of the W-group than to the N-like group;
*(3)*  *SCCs in the Q-like group are closer to the N-like group rather than the W-group*. In the Q-like group, there is glutamine at position 170 (Q178 in 6V4W; Table 1; position numbering according to 4UA6), which is not seen in the N-like group, but, similarly to the N-like group, this glutamine at position 170 interacts with the residue at the position preceding the catalytic nucleophile (for reference, see the interaction between N170 and C69 in the N-like group, Figure 3A). As a result, in the Q-like group, the intermediate glutamate appears (E167 in 6V4W; Table 1), which fulfills the role of E166 in N-like proteins (Figure 3A);
*(4)*  *NucBase-Omega zones are different, NucBase-Oxy zones are similar*. In β-lactamase/D-Ala carboxypeptidase family proteins, the main structural differences between SCCs in different groups are situated in their respective NucBase-Omega zones. At the same time, the NucBase-Oxy zones of the W-, G-, G-like, and Q-like groups are similar.

### 2.5. SCC in Proteins of the Glutaminase Family (Example: Glutaminase 1)

The glutaminase family is one of the three families within the β-lactamase/transpeptidase-like superfamily [14]. This family includes five representative structures that form only one group according to our classification, the “C-group” (Figure 4C, Table 1).

The glutaminase 1 structure (PDB ID: 1U60, 1.61 Å resolution) [57] is the representative structure of this family and group. In the NucBase-Omega zone, the CA atom of Cys_196_ from the Ω-subzone (shown in green in Figure 4C) interacts with the main-chain oxygen atom of an amino acid from the NucBase zone (Glu65 in Figure 4C) by means of a weak hydrogen bond, as also seen in proteins of the G- and G-like groups (Table S2, column II). However, the zone-forming contact between the Ω-subzone and Lys_69_ (the catalytic base in glutaminase 1) is formed differently when compared with the G- and G-like groups (Figure 4C vs. Figure 3C,D). In glutaminase 1, the donor of the intermediary atom is several residues away from the rest of the Ω-loop (Tyr_192_ in Figure 4C).

Another feature of this group of proteins is the presence of a hydrophobic amino acid at position 130 of the A-tripeptide (Leu115 in glutaminase 1; position numbering according to CTX-M-14). At the same time, an intermediary residue Tyr_244_ is found to interact with the Oxy subzone (Figure 4C; Table S2, column VIII) unlike the G- and G-like groups that have tyrosine at position 130 of the A-tripeptide. The SCC in proteins from the C-group consists of 17 amino acids (Table 1, row 24).

### 2.6. SCC in Proteins of the Dac-like Family (Example: D-Alanyl-D-Alanine Carboxypeptidase DacB)

The SCC of the D-alanyl-D-alanine carboxypeptidase DacB (PDB ID: 2EX2, R = 1.55 Å) [58] (Figure 4D, Table 1) is the representative structure for five proteins that form the G-group of the Dac-like family proteins (line 25 in Table 1), which is similar to the G-group of D-alanyl-D-alanine carboxypeptidases (line 11 in Table 1). However, there are two differences between the SCCs of the Dac-like and standard D-alanyl-D-alanine carboxypeptidases. The first difference is the presence of a serine instead of tyrosine at position 130 of the A-tripeptide (Ser306 in Figure 4D; position numbering according to CTX-M-14). The second difference is the extension of the NucBase segment by one residue due to the presence of a proline at the N-terminus. Despite these differences, both Dac-like and standard D-alanyl-D-alanine carboxypeptidases belong to the same group type (the G-group).

### 2.7. Overall Structural Comparison Between Groups of the β-Lactamase/Transpeptidase-like Superfamily Proteins and Molecular Function

Now that all the 3D structures analyzed in this study have been described and grouped (Table 1), it makes sense to structurally align the proteins within one group and between different groups and compare the corresponding RMSD values. The results are shown in Table S3. The overall structural comparison shows that the RMSD values within one group predominantly range between 2 Å and 3 Å, and the RMSD values between the groups range between 3 Å and 4 Å, which is in line with the grouping result. Unfortunately, this does not correlate the alignment of the SCCs with the overall structures. The SCC-based structural alignment using the software available to us does not look credible enough because of the significant fragmentation. Therefore, we used semi-manual identification of structurally equivalent residues and interactions between them.

Also, looking at the identified groups of β-lactamase/transpeptidase-like superfamily proteins, we have tabulated the molecular function and optimal pH where possible (Table S4). It is not surprising that the proteins within one group have a similar molecular function. However, the G-like group is the exception. Not only do the proteins within this group have different functions, but it also contains penicillin-binding protein 1b (PDB ID: 2BG1), which has multiple functions. Moreover, the multitude of functions of 2BG1 coincides with the multitude of functions of the representative protein of the G(Dac-like)-group, the D-alanyl-D-alanine carboxypeptidase DacB (PDB ID: 2EX2), showing that their cores work in a similar way (Table S4).

## 3. Materials and Methods

The Protein Data Bank (PDB, http://www.rcsb.org/; accessed on 10 October 2024 [5]) and the Structural Classification of Proteins (SCOP) database (https://www.ebi.ac.uk/pdbe/scop/; accessed on 10 October 2024 [14]) were used to retrieve 211 representative structures of proteins from the β-lactamase/transpeptidase-like superfamily (SCOP ID: 3001604).

Structure visualization and structural analysis of interactions (hydrogen bonds, non-polar interactions, and other weak interactions) were performed using Maestro software v.1.1 (Schrödinger Release 2023-1: Schrödinger, LLC, New York, NY, USA, 2021; http://www.schrodinger.com/; accessed on 10 October 2024). The class of β-lactamases was determined using the Ambler Classification system and the β-lactamase database (BLDT, http://bldb.eu/S-BLDB.php; accessed on 10 October 2024) [26]. Identification of protein residues involved in contact with a ligand was carried out using the PDBsum database (https://www.ebi.ac.uk/thornton-srv/databases/pdbsum/; accessed on 10 October 2024 [33]) and the Ligplot tool [34].

Weak hydrogen bonds were identified using criteria from [32]. The π–π stacking and other similar interactions were analyzed using the Residue Interaction Network Generator (RING, https://ring.biocomputingup.it/; accessed on 10 October 2024) [59]. Figures were drawn with MOLSCRIPT [60] and the PyMOL molecular graphics software (https://pymol.org/; accessed on 10 October 2024).

## 4. Conclusions

Structural studies of the catalytic sites of 199 proteins from the superfamily of β-lactamase/transpeptidase-like proteins revealed the similarities and differences among the Structural Catalytic Cores (SCCs) in these proteins, which consist of three distinct zones: the NucBase-Oxy zone, the NucBase-Omega zone, and the A-tripeptide link. The NucBase-Oxy zone is formed by the NucBase hexapeptide, containing a catalytic nucleophile and a catalytic base, and the Oxy tripeptide, followed by the amino acid that forms the oxyanion hole (Oxy tetrapeptide). In the process of constructing the NucBase-Omega zone, a structural requirement for localizing the omega(Ω)-subzone was formulated.

There are two ways in which the NucBase-Oxy zone and the NucBase-Omega zone join together to construct the SCCs. The first is when the amino acid at position 170 (position numbering according to CTX-M-14) of the Ω-subzone contacts simultaneously the nuc-1 residue from the NucBase hexapeptide and the Oxy hexapeptide. This variant is observed in the N-like group of proteins (class A β-lactamases). The second is when the same amino acid at position 170 (position numbering according to CTX-M-14) of the Ω-subzone contacts only the nuc-1 residue of the NucBase hexapeptide and nothing else. The second variant is observed in all of the other representative proteins.

If we consider both the type of amino acid at position 170 of the Ω-subzone and the nature of its interaction with the NucBase-Oxy zone, then all proteins of the β-lactamase/transpeptidase-like superfamily can be divided into seven different SCC groups, which further can be divided into 24 subgroups according to the key residue of the A-tripeptide. The proposed structural classification of the β-lactamase/transpeptidase-like proteins can be easily expanded to accommodate all new structures by using the approach proposed herein.

## Data Availability

All data supporting the reported results can be found in the Supplementary Materials.

## Supplementary Materials

The following supporting information can be downloaded at https://www.mdpi.com/article/10.3390/molecules30092019/s1: Table S1. Conserved geometric parameters (distance and angle) of contacts in 25 NucBase-Oxy zones of the β-lactamase/transpeptidase-like superfamily proteins. Table S2. Conserved geometric parameters (distance and angle) of contacts in 25 NucBase-Omega and BaseAlpha zones of the β-lactamase/transpeptidase-like superfamily proteins. Table S3. Pairwise root mean square deviation (RMSD) of atomic positions in 25 β-lactamase/transpeptidase-like superfamily representative proteins [61]. Table S4. Molecular function of the 25 β-lactamase/transpeptidase-like superfamily representative proteins [62].
