# Supplementary material for: Alpha and Omega Classification of β-Lactamase/Transpeptidase-like Superfamily Proteins Based on the Comparison of Their Structural Catalytic Cores
Source: Molecules. 2025 Apr 30;30(9):2019. doi: 10.3390/molecules30092019 (PMC12073871; doi:10.3390/molecules30092019)
Supplement: Supplementary file 1 [file molecules-30-02019-s001.zip › molecules-3571224-supplementary.pdf]

Supplementary materials

Alpha and Omega Classification of Beta-Lactamase/Transpeptidase-like Superfamily Proteins Based on Comparison of their Structural Catalytic Cores.

Alexander I. Denesyuk[1], Konstantin Denessiouk[1], Mark S. Johnson[1], Vladimir N. Uversky[2]

[1] Structural Bioinformatics Laboratory, Biochemistry, InFLAMES Research Flagship Center, Faculty of Science and Engineering, Åbo Akademi University, Turku 20520, Finland

[2] Department of Molecular Medicine and USF Health Byrd Alzheimer's Research Institute, Morsani College of Medicine, University of South Florida, Tampa, FL 33612, USA

Table S1. Conserved geometric parameters (distance and angle) of contacts in 25 NucBase-Oxy zones of the beta-lactamase/transpeptidase-like superfamily proteins.

N	PDB	R (Å)	Protein	I	II	III	IV	V	VI	Oxy	Sum
					Superfamily: beta-lactamase/transpeptidase-like<br>Family: beta-lactamase/D-Ala carboxypeptidase						
					N-like group (Class A)						79
					SNN subgroup						67
1	4UA6_A	0.79	Beta-lactamase CTX-M-14	O/S$_{70}$-N/K$_{73}$ 3.0	O/S$_{70}$-CE/K$_{234}$ 3.5 (2.5) 156°	NZ/K$_{234}$-O/T$_{235}$ 2.8	O/M$_{68}$-CA/G$_{236}$ 4.5 (3.5) 176°	O/M$_{68}$-N/T$_{71}$ 3.1<br>O/M$_{68}$-OG1/T$_{71}$ 2.8	N/G$_{236}$-O/N$_{245}$ 2.9<br>O/G$_{236}$-N/N$_{245}$ 2.8	N/S$_{70}$<br>N/S$_{237}$	
					SNS subgroup						5
2	5F82_A	0.96	Carbapenemase GES-5	O/S$_{64}$-N/K$_{67}$ 3.0	O/S$_{64}$-CE/K$_{229}$ 3.4 (2.4) 162°	NZ/K$_{229}$-O/T$_{230}$ 2.8	O/M$_{62}$-CA/G$_{231}$ 4.5 (3.5) 173°	O/M$_{62}$-N/T$_{65}$ 3.2<br>O/M$_{62}$-OG1/T$_{65}$ 2.7	N/G$_{231}$-O/N$_{239}$ 2.9<br>O/G$_{231}$-N/N$_{239}$ 2.9	N/S$_{64}$<br>N/T$_{232}$	
					SNG subgroup						2
3	2QPN_A	1.10	Carbapenemase GES-1	O/S$_{64}$-N/K$_{67}$ 3.0	O/S$_{64}$-CE/K$_{229}$ 3.3 (2.3) 161°	NZ/K$_{229}$-O/T$_{230}$ 2.8	O/M$_{62}$-CA/G$_{231}$ 4.9 (3.8) 174°	O/M$_{62}$-N/T$_{65}$ 3.1<br>O/M$_{62}$-OG1/T$_{65}$ 2.7	N/G$_{231}$-O/N$_{239}$ 2.9<br>O/G$_{231}$-N/N$_{239}$ 2.9	N/S$_{64}$<br>N/T$_{232}$	
					SSN subgroup						2
4	7DDM_A	1.20	Beta-lactamase PenA39	O/S$_{70}$-N/K$_{73}$ 3.0	O/S$_{70}$-CE/K$_{234}$ 3.3 (2.3) 151°	NZ/K$_{234}$-O/T$_{235}$ 2.8	O/F$_{68}$-CA/G$_{236}$ 4.5 (3.4) 173°	O/F$_{68}$-N/T$_{71}$ 3.1<br>O/F$_{68}$-OG1/T$_{71}$ 2.8	N/G$_{236}$-O/N$_{245}$ 3.0<br>O/G$_{236}$-N/N$_{245}$ 2.8	N/S$_{70}$<br>N/T$_{237}$	
					SGN subgroup						3
5	5NJ2_A	1.19	Beta-lactamase BlaC	O/S$_{70}$-N/K$_{73}$ 3.0	O/S$_{70}$-CE/K$_{236}$ 3.4 (2.4) 152°	NZ/K$_{236}$-O/T$_{235}$ 2.9	O/F$_{68}$-CA/G$_{238}$ 4.5 (3.4) 173°	O/F$_{68}$-N/T$_{71}$ 3.1<br>O/F$_{68}$-OG1/T$_{71}$ 2.8	N/G$_{238}$-O/N$_{246}$ 3.0<br>O/G$_{238}$-N/N$_{246}$ 2.7	N/S$_{70}$<br>N/T$_{239}$	
					W-group (Class D)						45
					SVW subgroup						36
6	5IY2_B	1.15	Beta-lactamase OXA-143	O/S$_{81}$-N/KCX$_{84}$ 2,9	O/S$_{81}$-CE/K$_{218}$ 3.6 (2.6) 146°	NZ/K$_{218}$-O/S$_{219}$ 2.9	O/P$_{79}$-CA/G$_{220}$ 4.6 (3.6) 157°	O/P$_{79}$-N/T$_{82}$ 3.2<br>O/P$_{79}$-OG1/T$_{82}$ 2.7	N/G$_{220}$-O/W$_{231}$ 2.9<br>O/G$_{220}$-N/W$_{231}$ 2.8	N/S$_{81}$<br>N/W$_{221}$	
					SIW subgroup						5
7	6W5E_A	1.30	Beta-lactamase BSU-2	O/S$_{101}$-N/KCX$_{104}$ 3.0	O/S$_{101}$-CE/K$_{239}$ 3.4 (2.3) 162°	NZ/K$_{239}$-O/T$_{240}$ 2.9	O/P$_{99}$-CA/G$_{241}$ 4.6 (3.5) 167°	O/P$_{99}$-N/T$_{102}$ 3.3<br>O/P$_{99}$-OG1/T$_{102}$ 2.7	N/G$_{241}$-O/W$_{251}$ 3.0<br>O/G$_{241}$-N/W$_{251}$ 2.8	N/S$_{101}$<br>N/T$_{242}$	
					SLW subgroup						4
8	6N1N_A	1.60	Beta-lactamase STD-1	O/S$_{65}$-N/KCX$_{68}$ 3.0	O/S$_{65}$-CE/K$_{203}$ 3.6 (2.7) 152°	NZ/K$_{203}$-O/T$_{204}$ 2.9	O/P$_{63}$-CA/G$_{205}$ 4.4 (3.5) 165°	O/P$_{63}$-N/T$_{66}$ 3.2<br>O/P$_{63}$-OG1/T$_{66}$ 2.8	N/G$_{205}$-O/W$_{216}$ 3.2<br>O/G$_{205}$-N/W$_{216}$ 2.8	N/S$_{65}$<br>N/W$_{206}$	
					W-group						5
					SNW subgroup						4
9	2IWB_A	1.80	Methicillin resistance mecR1 protein	O/S$_{391}$-N/K$_{394}$ 2.9	O/S$_{391}$-CE/K$_{528}$ 3.5 (2.5) 160°	NZ/K$_{528}$-O/T$_{529}$ 2.8	O/P$_{389}$-CA/G$_{530}$ 4.2 (3.2) 163°	O/P$_{389}$-N/T$_{392}$ 3.0<br>O/P$_{389}$-OG1/T$_{392}$ 2.7	N/G$_{530}$-O/W$_{542}$ 2.9<br>O/G$_{530}$-N/W$_{542}$ 2.8	N/S$_{391}$<br>N/T$_{531}$	
					STW subgroup						1
10	1NRF_A	2.50	Regulatory protein BlaR1	O/S$_{402}$-N/K$_{405}$ 3.2	O/S$_{402}$-CD/K$_{539}$ 2.9 (2.1) 127°	NZ/K$_{539}$-O/T$_{540}$ 3.2	O/P$_{400}$-CA/G$_{541}$ 4.7 (3.7) 154°	O/P$_{400}$-N/T$_{403}$ 3.1<br>O/P$_{400}$-OG1/T$_{403}$ 2.6	N/G$_{541}$-O/W$_{553}$ 2.8<br>O/G$_{541}$-N/W$_{553}$ 2.7	N/S$_{402}$<br>N/T$_{542}$	
					G-group						23
					YNG subgroup						4

11	1YQS_A	1.05	D-alanyl-D-alanine carboxypeptidase	O/S$_{62}$-N/K$_{65}$ 3.0	O/S$_{62}$-π/H$_{298}$ 3.6	ND1/H$_{298}$-O/T$_{299}$ 2.8	O/V$_{60}$-CA/G$_{300}$ 3.3 (2.3) 146°	O/V$_{60}$-N/V$_{63}$ 3.2	N/G$_{300}$-O/T$_{307}$ 2.8<br>O/G$_{300}$-N/T$_{307}$ 2.8	N/S$_{62}$<br>N/T$_{301}$	
					SNG subgroup						17
12	5ZQA_A	1.55	Lmo2812 protein	O/S$_{58}$-N/K$_{61}$ 2.9	O/S$_{58}$-CE/K$_{222}$ 3.7 (2.7) 161°	NZ/K$_{222}$-O/T$_{223}$ 2.9	O/I$_{56}$-CA/G$_{224}$ 4.8 (3.8) 165°	O/I$_{56}$-N/L$_{59}$ 3.0	N/G$_{224}$-O/C$_{232}$ 3.0<br>O/G$_{224}$-N/C$_{232}$ 2.8	N/S$_{58}$<br>N/F$_{225}$	
					SCG subgroup						1
13	1ES5_A	1.40	DD-transpeptidase	O/S$_{35}$-N/K$_{38}$ 2.9	O/S$_{35}$-CE/K$_{213}$ 3.7 (2.7) 150°	NZ/K$_{213}$-O/T$_{214}$ 2.9	O/T$_{33}$-CA/G$_{215}$ 4.5 (3.5) 160°	O/T$_{33}$-N/T$_{36}$ 3.4<br>O/T$_{33}$-OG1/T$_{36}$ 2.7	N/G$_{215}$-O/C$_{223}$ 2.9<br>O/G$_{215}$-N/C$_{223}$ 2.8	N/S$_{35}$<br>N/A$_{216}$	
					YSG subgroup						1
14	1WYB_A	1.80	6-aminohexanoate-dimer hydrolase	O/S$_{112}$-N/K$_{115}$ 2.9	O/S$_{112}$-HOH$_{393}$ 2.8<br>HOH$_{393}$-CA/G$_{342}$ 3.2 (2.5) 119°	HOH$_{393}$-O/I$_{343}$ 3.5	O/L$_{110}$-CA/G$_{344}$ 4.0 (2.9) 169°	O/L$_{110}$-N/V$_{113}$ 3.1	N/G$_{344}$-O/Q$_{348}$ 2.8<br>O/G$_{344}$-N/Q$_{348}$ 2.9	N/S$_{112}$<br>N/I$_{345}$	
					G-like group						39
					YNY subgroup (Class C)						33
15	6FM6_A	1.05	Beta-lactamase TRU-1	O/S$_{62}$-N/K$_{65}$ 2.9	O/S$_{62}$-CE/K$_{312}$ 3.6 (2.5) 158°	NZ/K$_{312}$-O/T$_{313}$ 2.8	O/I$_{60}$-CA/G$_{314}$ 3.3 (2.3) 156°	O/I$_{60}$-N/V$_{63}$ 3.1<br>O/I$_{60}$-CG2/V$_{63}$ 2.6 (2.9) 125°	N/G$_{314}$-O/A$_{321}$ 3.0<br>O/G$_{314}$-N/A$_{321}$ 2.8	N/S$_{62}$<br>N/S$_{315}$	
					YNA subgroup						1
16	1EI5_A	1.90	D-aminopeptidase	O/S$_{62}$-N/K$_{65}$ 3.1	O/S$_{62}$-π/H$_{287}$ 3.8	CD2/H$_{287}$-O/G$_{288}$ 2.7 (1.7) 156°	O/I$_{60}$-CA/G$_{289}$ 3.9 (2.9) 171°	O/I$_{60}$-N/V$_{63}$ 3.0	N/G$_{289}$-O/C$_{296}$ 2.9<br>O/G$_{289}$-N/C$_{296}$ 3.0	N/S$_{62}$<br>N/A$_{290}$	
					YLA subgroup						2
17	1CI9_A	1.80	Esterase EstB	O/S$_{75}$-N/K$_{78}$ 3.0	O/S$_{75}$-CD1/W$_{348}$ 4.3 (3.3) 160°	CD1/W$_{348}$-O/G$_{349}$ 3.7 (3.2) 112°	O/L$_{73}$-CA/G$_{350}$ 4.2 (3.2) 157°	O/L$_{73}$-N/V$_{76}$ 2.9	N/G$_{350}$-O/H$_{354}$ 2.9<br>O/G$_{350}$-N/H$_{354}$ 3.1	N/S$_{75}$<br>N/V$_{351}$	
					YHQ subgroup						1
18	4IVK_A	1.80	Carboxylesterase	O/S$_{100}$-N/K$_{103}$ 3.0	O/S$_{100}$-CD1/W$_{381}$ 3.6 (2.6) 150°	CD1/W$_{381}$-O/G$_{382}$ 3.6 (3.2) 100°	O/I$_{98}$-CA/G$_{383}$ 3.5 (2.6) 147°	O/I$_{98}$-N/M$_{101}$ 2.9	N/G$_{383}$-O/T$_{387}$ 2.8<br>O/G$_{383}$-N/G$_{386}$ 3.0	N/S$_{100}$<br>N/A$_{384}$	
					YPH subgroup						1
19	6KJC_A	2.30	Lovastatin esterase	O/S$_{57}$-N/K$_{60}$ 2.9	O/S$_{57}$-HOH$_{600}$ 3.2<br>HOH$_{600}$-CD1/W$_{344}$ 3.5 (2.7) 133°	CD1/W$_{344}$-O/G$_{345}$ 3.4 (2.9) 113°	O/L$_{55}$-CA/G$_{346}$ 3.6 (2.6) 148°	O/L$_{55}$-N/A$_{58}$ 3.0	N/G$_{346}$-O/L$_{350}$ 2.9<br>O/G$_{346}$-N/L$_{350}$ 3.2	N/S$_{57}$<br>N/G$_{347}$	
					SNM subgroup						1
20	2BG1_A	1.90	Penicillin-binding protein 1b	O/S$_{460}$-N/K$_{463}$ 3.3	O/S$_{460}$-CE/K$_{651}$ 3.9 (3.1) 132°	NZ/K$_{651}$-O/T$_{652}$ 2.8	O/P$_{458}$-CA/G$_{653}$ 4.2 (3.3) 142°	O/P$_{458}$-N/T$_{461}$ 3.2<br>O/P$_{458}$-OG1/T$_{461}$ 2.5	N/G$_{653}$-O/W$_{662}$ 2.8<br>O/G$_{653}$-N/W$_{662}$ 2.9	N/S$_{460}$<br>N/T$_{654}$	
					Q-like group (Class A)						6
					SNQ subgroup						5
21	6V4W_A	1.29	Beta-lactamase CPA-1	O/S$_{68}$-N/K$_{71}$ 3.0	O/S$_{68}$-CE/K$_{235}$ 3.4 (2.4) 153°	NZ/K$_{235}$-O/T$_{236}$ 2.9	O/M$_{66}$-CA/G$_{237}$ 4.0 (2.9) 169°	O/M$_{66}$-N/V$_{69}$ 3.1	N/G$_{237}$-O/N$_{250}$ 3.0<br>O/G$_{237}$-N/N$_{250}$ 2.8	N/S$_{68}$<br>N/S$_{238}$	
					SNT subgroup						1
22	5TFQ_A	1.07	Beta-lactamase HGB-2	O/S$_{48}$-N/K$_{73}$ 3.0	O/S$_{48}$-CE/K$_{215}$ 3.3 (2.3) 149°	NZ/K$_{215}$-O/T$_{216}$ 2.8	O/L$_{46}$-CA/G$_{217}$ 4.3 (3.2) 178°	O/L$_{46}$-N/V$_{49}$ 3.0	N/G$_{217}$-O/N$_{231}$ 3.1<br>O/G$_{217}$-N/N$_{231}$ 2.8	N/S$_{48}$<br>N/S$_{218}$	
					Inactive beta-lactamase group						2
					GKN subgroup						2

23	5IHV_A	1.10	Beta-lactamase *B. ambifaria* MC40-6	O/G_{47}-N/A_{50} 3.0	O/G_{47}-CE/K_{211} 3.4 (2.4) 160°	NZ/K_{211}-O/A_{212} 2.7	O/L_{45}-CA/G_{213} 4.7 (3.6) 176°	O/L_{45}-N/T_{48} 3.1<br>O/L_{45}-$OG1/T_{48}$ 2.7	N/G_{213}-O/T_{221} 2.7<br>O/G_{213}-N/T_{221} 2.9	N/G_{47}<br>N/T_{214}	
					Family: glutaminase						
					C-group						5
					ONC subgroup						5
24	1U60_A	1.61	Glutaminase 1	O/S_{66}-N/K_{69} 3.0	O/S_{66}-CE/K_{259} 3.3 (2.2) 161°	NZ/K_{259}-O/S_{260} 2.7	O/L_{64}-CA/G_{261} 4.3 (3.2) 165°	O/L_{64}-N/I_{67} 3.2	N/G_{261}-O/G_{265} 3.2<br>O/G_{261}-N/G_{264} 3.1	N/S_{66}<br>N/V_{262}	
					Family: Dac-like						
					G(Dac-like)-group						5
					SNG subgroup						5
25	2EX2_A	1.55	D-alanyl-D-alanine carboxypeptidase DacB	O/S_{62}-N/K_{65} 3.0	O/S_{62}-CE/K_{417} 3.6 (2.6) 148°	NZ/K_{417}-O/T_{418} 2.8	O/P_{60}-CA/G_{419} 4.3 (3.2) 162°	O/P_{60}-N/T_{63} 3.1<br>O/P_{60}-$OG1/T_{63}$ 2.7	N/G_{419}-O/N_{426} 2.9<br>O/G_{419}-N/N_{426} 2.7	N/S_{62}<br>N/G_{419}	

Table S2. Conserved geometric parameters (distance and angle) of contacts in 25 NucBase-Omega and BaseAlpha zones of the beta-lactamase/transpeptidase-like superfamily proteins.

N	PDB	Protein	I	II	III	IV	V	VI	VII	VIII
Superfamily: beta-lactamase/transpeptidase-like<br>Family: beta-lactamase/D-Ala carboxypeptidase<br>N-like group (Class A)<br>SNN subgroup										
1	4UA6_A	Beta-lactamase CTX-M-14	ND2/N_{170}-OE2/E_{166} 2.7<br>OE1/E_{166}-NZ/K_{73} 2.9	OD1/N_{170}-CA/C_{69}<br>3.3 (2.6) 126°	O/N_{170} -N/D_{240} 3.1	N/M_{68}-O/D_{179} 3.0	N/R_{161}-O/T_{180} 3.1<br>O/R_{161}-N/T_{180} 3.4	N/C_{69}-HOH_{2167} 2.8<br>O/L_{169}-HOH_{2167} 2.7<br>O/D_{179}-HOH_{2167} 2.8	N/T_{171}-O/T_{168} 3.2	NZ/K_{234}-OG/S_{130} 2.8<br>NZ/K_{73}-OD1/N_{132} 2.8
SNS subgroup										
2	5F82_A	Carbapenemase GES-5	OG/S_{165}-OE2/E_{161} 2.8<br>OE1/E_{161}-CE/K_{67}<br>3.2 (2.5) 135°	OG/S_{165}-CA/G_{63}<br>3.4 (2.6) 143°	O/S_{165} -N/A_{234} 3.2	N/M_{62}-O/D_{174} 2.9	N/R_{156}-O/T_{175} 3.0<br>O/R_{156}-N/T_{175} 3.5	N/G_{63}-HOH_{498} 2.8<br>O/M_{164}-HOH_{498} 2.7<br>O/D_{174}-HOH_{498} 2.8	N/D_{166}-O/E_{163} 3.2	NZ/K_{229}-OG/S_{125} 2.9<br>NZ/K_{67}-OD1/N_{127} 2.7
SNG subgroup										
3	2QPN_A	Carbapenemase GES-1	CA/G_{165}-HOH_{390}<br>3.5 (2.6) 139°<br>HOH_{390}-OE2/E_{161} 3.1<br>OE1/E_{161}-CE/K_{67}<br>3.2 (2.5) 123°	CA/G_{165}-CA/G_{63}<br>4.9	O/G_{165} -N/A_{234} 3.3	N/M_{62}-O/D_{174} 2.9	N/R_{156}-O/T_{175} 2.9<br>O/R_{156}-N/T_{175} 3.5	N/G_{63}-HOH_{338} 2.8<br>O/M_{164}-HOH_{338} 2.9<br>O/D_{174}-HOH_{338} 2.9	N/D_{166}-O/E_{163} 3.0	NZ/K_{229}-OG/S_{125} 2.9<br>NZ/K_{67}-OD1/N_{127} 2.7
SSN subgroup										
4	7DDM_A	Beta-lactamase PenA39	ND2/N_{170}-OE1/E_{166} 3.0<br>OE2/E_{166}-NZ/K_{73} 3.0	OD1/N_{170}-CA/C_{69}<br>3.4 (2.6) 124°	O/N_{170} -N/D_{240} 3.2	N/F_{68}-O/D_{179} 3.1	N/R_{161}-O/T_{180} 3.1<br>O/R_{161}-N/T_{180} 3.3	N/C_{69}-HOH_{476} 2.8<br>O/L_{169}-HOH_{476} 2.8<br>O/D_{179}-HOH_{476} 2.8	N/T_{171}-O/E_{168} 3.4	NZ/K_{234}-OG/S_{130} 2.7<br>NZ/K_{73}-HOH_{464} 2.9<br>HOH_{464}-OG/S_{132} 2.8
SGN subgroup										
5	5NJ2_A	Beta-lactamase BlaC	ND2/N_{172}-OE2/E_{168} 2.8<br>OE1/E_{168} -CE/K_{73}<br>3.3 (2.6) 118°	OD1/N_{172}-CA/C_{69}<br>3.6 (2.7) 136°	O/N_{172} -N/D_{241} 3.3	N/F_{68}-O/D_{181} 3.0	N/R_{163}-O/T_{182} 2.9<br>O/R_{163}-N/T_{182} 3.1	N/C_{69}-HOH_{547} 2.8<br>O/L_{171}-HOH_{547} 2.7<br>O/D_{181}-HOH_{547} 2.9	N/R_{173}-O/E_{170} 3.0	NZ/K_{236}-OG/S_{128} 2.8<br>NZ/K_{73}-HOH_{544} 2.7<br>HOH_{544}-CA/G_{130}<br>3.2 (2.6) 112°
W-group (Class D)<br>SVW subgroup										
6	5IY2_B	Beta-lactamase OXA-143	NE1/W_{167}-OQ2/KCX_{84} 3.0	CD1/W_{167}-O/A_{80}<br>3.3 (2.4) 143°	O/W_{167}-N/A_{80} 2.9	CA/V_{78}-O/L_{172}<br>3.7 (2.7) 160°	N/N_{156}-O/K_{173} 2.9<br>OD1/N_{156}-N/K_{173} 3.2	CD/P_{79}-HOH_{303}<br>3.7 (2.6) 165°<br>O/F_{166}-HOH_{303} 2.4<br>O/L_{172}-HOH_{303} 2.7	N/L_{168}-O/N_{165} 3.0	NZ/K_{218}-OG/S_{128} 3.0<br>OQ2/KCX_{84}-CG2/V_{130}<br>3.6 (2.9) 120°
SIW subgroup										
7	6W5E_A	Beta-lactamase BSU-2	NE1/W_{188}-OQ2/KCX_{104} 3.0	CD1/W_{188}-O/Q_{100}<br>3.3 (2.4) 148°	O/W_{188}-N/Q_{100} 2.9<br>N/Q_{190}-OE1/Q_{100} 2.9<br>OE1/Q_{190}-NE2/Q_{100} 3.0	CA/T_{98}-O/L_{193}<br>3.4 (2.4) 153°	N/N_{177}-O/T_{194} 2.9<br>OD1/N_{177}-N/T_{194} 2.9	CD/P_{99}-HOH_{450}<br>3.9 (2.8) 162°<br>O/F_{187}-HOH_{450} 2.7<br>O/L_{193}-HOH_{450} 2.8	N/L_{189}-O/Q_{186} 2.9	NZ/K_{239}-OG/S_{149} 2.6<br>OQ2/KCX_{104}-CG1/I_{151}<br>3.2 (2.2) 167°
SLW subgroup										
8	6N1N_A	Beta-lactamase STD-1	NE1/W_{152}-OQ2/KCX_{68} 3.1	CD1/W_{152}-O/A_{64}<br>3.5 (2.7) 146°	O/W_{152}-N/A_{64} 3.0	CA/L_{62}-O/L_{157}<br>3.4 (2.5) 162°	N/N_{141}-O/K_{158} 2.9<br>OD1/N_{141}-N/K_{158} 3.0	CD/P_{63}-HOH_{510}<br>4.1 (3.1) 179°<br>O/F_{151}-HOH_{510} 2.8	N/I_{153}-O/S_{150} 3.1	NZ/K_{203}-OG/S_{113} 2.7<br>OQ2/KCX_{68}-CD1/L_{115}<br>3.6 (2.9) 129°

								O/L$_{157}$-HOH$_{510}$ 2.9		
W-group<br>SNW subgroup										
9	2IWB_A	Methicillin resistance mecR1 protein	NE1/W$_{477}$-HOH$_{2042}$ 3.6<br>HOH$_{2042}$-NZ/K$_{394}$ 3.1	CD1/W$_{477}$-O/N$_{390}$ 3.9 (3.2) 129°	O/W$_{477}$-N/N$_{390}$ 2.9<br>N/E$_{479}$-OD1/N$_{390}$ 3.1<br>OE2/E$_{479}$-ND2/N$_{390}$ 2.8	CA/S$_{388}$-O/L$_{482}$ 3.3 (2.3) 163°	N/N$_{467}$-O/K$_{483}$ 2.9<br>OD1/N$_{467}$-N/K$_{483}$ 2.9	CD/P$_{389}$-HOH$_{2115}$ 3.9 (2.8) 157°<br>O/Y$_{476}$-HOH$_{2115}$ 2.7<br>O/L$_{482}$-HOH$_{2115}$ 2.6	N/N$_{478}$-O/N$_{475}$ 3.1	NZ/K$_{528}$-OG/S$_{439}$ 2.9<br>NZ/K$_{394}$-OD1/N$_{441}$ 2.8
STW subgroup										
10	1NRF_A	Regulatory protein BlaR1	CZ3/W$_{488}$-NZ/K$_{405}$ 3.8	CE3/W$_{488}$-O/A$_{401}$ 3.5 (2.6) 138°	O/W$_{488}$-N/A$_{401}$ 2.8	CA/A$_{399}$-O/L$_{493}$ 3.6 (2.5) 173°	N/N$_{478}$-O/Q$_{494}$ 2.8<br>OD1/N$_{478}$-N/Q$_{494}$ 3.0	CD/P$_{400}$-HOH$_{738}$ 3.9 (2.8) 167°<br>O/Y$_{487}$-HOH$_{738}$ 2.7<br>O/L$_{493}$-HOH$_{738}$ 2.7	N/L$_{489}$-O/D$_{486}$ 2.7	NZ/K$_{539}$-OG/S$_{450}$ 2.8<br>NZ/K$_{405}$-OG1/T$_{452}$ 3.6
G-group<br>YNG subgroup										
11	1YQS_A	D-alanyl-D-alanine carboxy-peptidase	O/A$_{237}$-NZ/K$_{65}$ 3.1	CA/G$_{238}$-O/G$_{61}$ 3.3 (2.3) 150°	O/G$_{238}$-CA/G$_{61}$ 3.3 (2.5) 129°	N/V$_{60}$-O/V$_{240}$ 3.1	N/F$_{197}$-O/I$_{241}$ 2.9<br>O/F$_{197}$-N/I$_{241}$ 2.9	N/G$_{61}$-HOH$_{2012}$ 3.0<br>O/G$_{238}$-HOH$_{2012}$ 2.7<br>O/V$_{240}$-HOH$_{2012}$ 2.7	N/A$_{239}$-O/Q$_{235}$ 2.9	CE1/H$_{298}$-OH/Y$_{159}$ 3.7 (2.7) 155°<br>NZ/K$_{65}$-OD1/N$_{161}$ 3.0
SNG subgroup										
12	5ZQA_A	Lmo2812 protein	O/S$_{158}$-NZ/K$_{61}$ 3.0	CA/G$_{159}$-O/A$_{57}$ 3.4 (2.4) 152°	O/G$_{159}$-CA/A$_{57}$ 3.7 (2.7) 153°	N/I$_{56}$-O/A$_{167}$ 3.0	N/T$_{153}$-O/V$_{168}$ 3.0<br>O/T$_{153}$-N/V$_{168}$ 3.0	N/A$_{57}$-HOH$_{515}$ 2.9<br>HOH$_{515}$-HOH$_{418}$ 2.8<br>O/G$_{159}$-HOH$_{418}$ 2.6<br>O/A$_{167}$-HOH$_{515}$ 2.9	N/L$_{160}$-OG/S$_{158}$ 3.1	NZ/K$_{222}$-OG/S$_{118}$ 2.8<br>NZ/K$_{61}$-OD1/N$_{120}$ 2.9
SCG subgroup										
13	1ES5_A	DD-transpeptidase	O/D$_{143}$-CE/K$_{38}$ 3.7 (2.8) 138°	CA/G$_{144}$-O/G$_{34}$ 3.4 (2.3) 169°	O/G$_{144}$-CA/G$_{34}$ 4.4 (3.7) 124°	N/T$_{33}$-HOH$_{347}$ 3.1<br>HOH$_{347}$-O/N$_{150}$ 2.8	N/H$_{138}$-O/Y$_{151}$ 2.9<br>O/H$_{138}$-N/Y$_{151}$ 2.9	N/G$_{34}$-HOH$_{479}$ 3.0<br>O/G$_{144}$-HOH$_{479}$ 2.7<br>HOH$_{479}$-HOH$_{347}$ 2.7<br>O/A$_{150}$-HOH$_{347}$ 2.8	N/I$_{145}$-OD1/D$_{143}$ 2.9	NZ/K$_{213}$-OG/S$_{96}$ 2.9<br>NZ/K$_{38}$-SG/C$_{98}$ 2.8
YSG subgroup										
14	1WYB_A	6-aminohexanoate-dimer hydrolase	O/H$_{266}$-NZ/K$_{115}$ 3.1	CA/G$_{267}$-O/M$_{111}$ 3.3 (2.4) 147°	O/G$_{267}$-N/M$_{111}$ 3.4	N/L$_{110}$-O/V$_{269}$ 2.7	N/T$_{254}$-O/S$_{270}$ 3.0<br>O/T$_{254}$-N/S$_{270}$ 3.0	N/M$_{111}$-CG/L$_{109}$ 3.7<br>O/G$_{267}$- CD1/L$_{109}$ 3.5 (2.5) 141°<br>O/V$_{269}$- CD1/L$_{109}$ 3.6 (2.5) 169°	N/G$_{268}$-O/F$_{264}$ 2.9	CA/G$_{342}$-HOH$_{393}$ 3.2 (2.5) 119°<br>HOH$_{393}$-HOH$_{410}$ 3.3<br>HOH$_{410}$-OH/Y$_{215}$ 2.8<br>NZ/K$_{115}$-OG/S$_{217}$ 2.9
G-like group<br>YNY subgroup (Class C)										
15	6FM6_A	Beta-lactamase TRU-1	O/A$_{219}$-NZ/K$_{65}$ 2.8	CA/Y$_{220}$-O/G$_{61}$ 3.4 (2.4) 148°	O/Y$_{220}$-CA/G$_{61}$ 3.6 (2.8) 130°	N/I$_{60}$-O/I$_{222}$ 3.2	N/Y$_{186}$-O/K$_{223}$ 2.9<br>O/Y$_{186}$-N/K$_{223}$ 2.8	N/G$_{61}$-HOH$_{537}$ 2.9<br>O/Y$_{220}$-HOH$_{537}$ 2.7<br>O/I$_{222}$-HOH$_{537}$ 2.8	N/G$_{221}$-O/A$_{216}$ 2.8	NZ/K$_{312}$-OH/Y$_{148}$ 2.8<br>NZ/K$_{65}$-OD1/N$_{150}$ 2.7
YNA subgroup										
16	1EI5_A	D-aminopeptidase	O/D$_{225}$-NZ/K$_{65}$ 2.9	CA/A$_{226}$-O/C$_{61}$ 3.3 (2.3) 147°	N/G$_{227}$-HOH$_{531}$ 3.0<br>HOH$_{531}$-CA/C$_{61}$ 3.4 (2.8) 114°	N/I$_{60}$-O/I$_{228}$ 2.9	N/E$_{191}$-O/C$_{229}$ 3.0<br>O/E$_{191}$-N/C$_{229}$ 2.8	N/C$_{61}$-HOH$_{531}$ 3.0<br>N/G$_{227}$-HOH$_{531}$ 3.0<br>O/I$_{228}$-HOH$_{531}$ 2.9	N/C$_{229}$-O/E$_{191}$ 2.8	NE2/H$_{287}$-OH/Y$_{153}$ 3.3<br>NZ/K$_{65}$-ND2/N$_{155}$ 2.7
YLA subgroup										
17	1CI9_A	Esterase EstB	O/G$_{274}$-NZ/K$_{78}$ 2.8	CA/A$_{275}$-O/A$_{74}$ 3.3 (2.3) 147°	O/A$_{275}$-CA/A$_{74}$ 3.5 (2.5) 144°	N/L$_{73}$-O/M$_{277}$ 2.8	N/G$_{218}$-O/Y$_{278}$ 3.2<br>O/G$_{218}$-N/Y$_{278}$ 2.8	N/A$_{74}$-HOH$_{1050}$ 3.0<br>O/A$_{275}$-HOH$_{1050}$ 2.7<br>O/M$_{277}$-HOH$_{1050}$ 2.8	N/G$_{276}$-O/S$_{272}$ 2.8	NE1/W$_{348}$-OH/Y$_{181}$ 2.9<br>NZ/K$_{78}$-OH/Y$_{133}$ 2.8<br>OH/Y$_{133}$-CA/L$_{183}$ 4.3 (3.3) 150°
YHQ subgroup										
18	4IVK_A	Carboxylesterase	O/G$_{295}$-NZ/K$_{103}$ 2.8	CA/Q$_{296}$-O/Y$_{99}$ 3.5 (2.6) 138°	O/Q$_{296}$-CA/Y$_{99}$ 3.3 (2.2) 170°<br>CA/M$_{282}$-OH/Y$_{99}$ 3.7 (3.0) 123°	N/I$_{98}$-O/M$_{298}$ 3.0	N/G$_{255}$-O/V$_{299}$ 3.0<br>O/G$_{255}$-N/V$_{299}$ 2.8	N/Y$_{99}$-HOH$_{666}$ 2.9<br>O/Q$_{296}$-HOH$_{666}$ 2.8<br>O/M$_{298}$-HOH$_{666}$ 2.8	N/G$_{297}$-O/P$_{293}$ 2.9	NE1/W$_{381}$-OH/Y$_{218}$ 2.8<br>NZ/K$_{103}$-OH/Y$_{172}$ 2.8<br>OH/Y$_{172}$-CA/H$_{220}$ 4.7 (3.8) 144°
YPH subgroup										
19	6KJC_A	Lovastatin esterase	O/G$_{252}$-NZ/K$_{60}$ 2.9	CA/H$_{253}$-O/A$_{56}$	O/H$_{253}$-CA/A$_{56}$	N/L$_{55}$-O/L$_{255}$ 3.1	N/Q$_{207}$-O/F$_{256}$ 3.3	N/A$_{56}$-π/Y$_{54}$ 3.5	N/G$_{254}$-O/F$_{250}$ 2.9	NE1/W$_{344}$-OH/Y$_{170}$ 2.8

				3.3 (2.3) 154°	3.6 (2.6) 151°<br>O/H_{253}-OH/Y_{54} 2.6		O/Q_{207}-N/F_{256} 2.9	O/H_{253}-OH/Y_{54} 2.6<br>O/L_{255}-CD/Y_{54}<br>3.6 (2.7) 137°		NZ/K_{60}-OH/Y_{127} 3.3<br>OH/Y_{127}-CA/P_{172}<br>4.1 (3.1) 148°
SNM subgroup										
20	2BG1_A	Penicillin-binding protein 1b	O/P_{555}-CE/K_{403}<br>4.2 (3.6) 113°	CA/M_{556}-O/A_{459}<br>4.1 (3.7) 107°	O/M_{556}-CA/A_{459}<br>3.4 (2.5) 133°	CA/S_{457}-O/I_{560}<br>3.2 (2.2) 150°	N/E_{545}-HOH_{2197} 3.0<br>HOH_{2197}-HOH_{2218} 2.6<br>HOH_{2218}-O/E_{561} 2.6<br>O/E_{545}-HOH_{2196} 2.8<br>HOH_{2196}-N/E_{561} 3.0	N/A_{459}-OG/S_{457} 3.2<br>O/G_{557}-OG/S_{457} 2.8<br>O/I_{560}-OG/S_{457} 3.9	N/G_{557}-O/L_{554} 2.9	NZ/K_{651}-OG/S_{516} 2.9<br>NZ/K_{463}-OD1/N_{518} 3.1
Q-like group (Class A)<br>SNQ subgroup										
21	6V4W_A	Beta-lactamase CPA-1	OE1/Q_{177}-NE2/Q_{67} 2.9<br>OE1/Q_{67}-HOH_{403} 3.0<br>HOH_{403}-OE2/E_{167} 2.4<br>OE2/E_{167}-NZ/K_{71} 3.2	N/A	OE1/Q_{177}-NE2/Q_{67} 2.9	N/M_{66}-O/N_{180} 2.9	N/A_{162}-O/W_{181} 2.9<br>O/A_{162}-N/W_{181} 3.3	N/Q_{67}-HOH_{498} 2.8<br>O/Q_{177}-HOH_{498} 2.8<br>O/N_{180}-HOH_{498} 3.4	N/Y_{178}-O/K_{175} 3.0	NZ/K_{235}-OG/S_{131} 3.0<br>NZ/K_{71}-OD1/N_{133} 2.7
SNT subgroup										
22	5TFQ_A	Beta-lactamase HGB-2	CG2/T_{157}-CD1/L_{47} 3.9<br>CD1/L_{47}-HOH_{446}<br>3.5 (3.0) 111°<br>HOH_{446}-OE2/E_{148} 2.6<br>OE2/E_{148}-NZ/K_{51} 3.3	N/A	CG2/T_{157}-CD1/L_{47} 3.9	N/L_{46}-O/N_{160} 3.0	N/N_{143}-O/W_{161} 3.0<br>O/N_{143}-N/W_{161} 2.9	N/L_{47}-HOH_{472} 2.9<br>O/T_{157}-HOH_{472} 2.7<br>O/N_{160}-HOH_{472} 2.7	N/A	NZ/K_{215}-OG/S_{112} 2.8<br>NZ/K_{51}-OD1/N_{114} 2.7
Inactive beta-lactamase group<br>GKN subgroup										
23	5IHV_A	Beta-lactamase<br>*B. ambifaria*<br>MC40-6	ND2/N_{147}-OE1/E_{143} 2.8<br>OE2/E_{143}-O2/EDO_{302} 2.7<br>C1/EDO_{302}-CB/A_{50} 4.2<br>OE1/E_{143}-NZ/K_{109} 2.9	OD1/N_{147}-CA/C_{46}<br>3.2 (2.4) 127°	O/N_{147}-N/G_{216} 2.9	N/L_{45}-O/D_{156} 2.9	N/R_{138}-O/T_{157} 3.0<br>O/R_{138}-N/T_{157} 3.0	N/C_{46}-HOH_{484} 2.8<br>O/L_{146}-HOH_{484} 2.8<br>O/D_{156}-HOH_{484} 2.8	N/L_{148}-O/L_{145} 3.0	NZ/K_{211}-O1/EDO_{302} 2.8<br>O1/EDO_{302}-CA/G_{107}<br>3.5 (2.7) 132°<br>CB/A_{50}-C1/EDO_{302} 4.2<br>O2/EDO_{302}-CG/K_{109}<br>4.2 (2.5) 143°
Family: glutaminase<br>C-group<br>ONC subgroup										
24	1U60_A	Glutaminase 1	SG/C_{196}-OH/Y_{192} 3.5<br>OH/Y_{192}-CE/K_{69}<br>3.1 (2.4) 119°	CA/C_{196}-O/E_{65}<br>3.7 (2.7) 153°	O/C_{196}-N/E_{65} 2.9<br>OE2/E_{161}-OE2/E_{65} 2.7	N/L_{64}-O/T_{198} 2.9	N/A_{152}-HOH_{1303} 3.3<br>HOH_{1303}-O/L_{199} 2.7<br>O/A_{152}-N/L_{199} 3.1	O/C_{196}-N/E_{65} 2.9<br>O/T_{198} -N/L_{64} 2.9	N/S_{197}-O/R_{194} 2.9	NZ/K_{259}-OH/Y_{244} 2.8<br>OH/Y_{244}-O/L_{115} 3.7<br>NZ/K_{69}-OD1/N_{117} 2.5
Family: Dac-like<br>G(Dac-like)-group<br>SNG subgroup										
25	2EX2_A	D-alanyl-D-alanine carboxypeptidase DacB	O/S_{357}-NZ/K_{65} 2.9	CA/G_{358}-O/A_{61}<br>3.2 (2.2) 161°	O/G_{358}-CA/A_{61}<br>3.5 (2.5) 148°	CA/L_{59}-O/N_{363}<br>4.0 (2.9) 168°	N/I_{352}-O/L_{364} 2.8<br>O/I_{352}-N/L_{364} 3.1	N/A_{61}-HOH_{1005} 3.0<br>O/G_{358}-HOH_{1005} 2.7<br>OD1/N_{363}-HOH_{1005}<br>3.4<br>CD/P_{60}-HOH_{1005}<br>3.5 (2.6) 133°	N/L_{359}-OG/S_{357} 3.2	NZ/K_{417}-OG/S_{306} 2.9<br>NZ/K_{65}-OD1/N_{308} 2.7

N/A–Not Available.

Table S3. Pairwise root mean square deviation (RMSD) of atomic positions in 25 beta-lactamase/transpeptidase-like superfamily representative proteins.

				N-like group				
		5F82_A	2QPN_A	7DDM_A	5NJ2_A			
1	4UA6_A	1.7 [1]	1.7	1.0	1.2			
				W-group				
		6W5E_A	6N1N_A	2IWB_A	1NRF_A			
2	5IY2_B	1.9	1.7	2.2	1.8			
				G-group				
		5ZQA_A	1ES5_A	1WYB_A				
3	1YQS_A	2.6	2.7	2.7				
				G-like group				
		1EI5_A	1CI9_A	4IVK_A	6KJC_A	2BG1_A		
4	6FM6_A	2.7	2.8	2.8	3.1	3.7		
				Q-like group				
		5TFQ_A						
5	6V4W_A	1.5						
				Between N-like group and other groups				
		W-: 5IY2_B	G-: 1YQS_A	G-like: 6FM6_A	Q-like: 6V4W_A	Inactive: 5IHV_A	C-: 1U60_A	G(Dac-like)-: 2EX2_A
6	4UA6_A	3.2	3.0	3.2	1.9	1.0	3.7	3.3
				Between W-group and other groups				
		G-: 1YQS_A	G-like: 6FM6_A	Q-like: 6V4W_A	Inactive: 5IHV_A	C-: 1U60_A	G(Dac-like)-: 2EX2_A	
7	5IY2_B	3.2	3.1	3.1	3.4	3.2	3.5	
				Between G-group and other groups				
		G-like: 6FM6_A	Q-like: 6V4W_A	Inactive: 5IHV_A	C-: 1U60_A	G(Dac-like)-: 2EX2_A		
8	1YQS_A	2.8	2.7	2.7	3.2	3.6		
				Between G-like group and other groups				
		Q-like: 6V4W_A	Inactive: 5IHV_A	C-: 1U60_A	G (Dac-like)-: 2EX2_A			
9	6FM6_A	3.2	3.2	3.5	3.3			
				Between Q-like group and other groups				
		Inactive: 5IHV_A	C: 1U60_A	G(Dac-like)-: 2EX2_A				
10	6V4W_A	2.1	3.5	2.7				
				Between inactive beta-lactamase group and other groups				
		C: 1U60_A	G (Dac-like): 2EX2_A					
11	5IHV_A	3.6	3.3					
				Between C- and G(Dac-like)-groups				
		G(Dac-like)-: 2EX2_A						

12	1U60_A	3.3

[1] Values RMSD were obtained from the Dali server: http://ekhidna2.biocenter.helsinki.fi/dali/. Holm L, Laiho A, Törönen P, Salgado M. DALI shines a light on remote homologs: One hundred discoveries. Protein Sci. 2023; 32(1):e4519. doi: 10.1002/pro.4519.

Table S4. Molecular function of the 25 beta-lactamase/transpeptidase-like superfamily representative proteins.

N	PDB ID	Protein	Molecular function
		Superfamily: beta-lactamase/transpeptidase-like	
		Family: beta-lactamase/D-Ala carboxypeptidase	
		N-like group (Class A) (79)	
		SNN subgroup (67)	
1	4UA6_A	Beta-lactamase CTX-M-14	beta-lactamase activity [1]
		SNS subgroup (5)	
2	5F82_A	Carbapenemase GES-5	beta-lactamase activity
		SNG subgroup (2)	
3	2QPN_A	Carbapenemase GES-1	beta-lactamase activity, pH [2] 7.0
		SSN subgroup (2)	
4	7DDM_A	Beta-lactamase PenA39	beta-lactamase activity
		SGN subgroup (3)	
5	5NJ2_A	Beta-lactamase BlaC	beta-lactamase activity, pH 6.4-7.5
		W-group (Class D) (45)	
		SVW subgroup (36)	
6	5IY2_B	Beta-lactamase OXA-143	penicillin binding
		SIW subgroup (5)	
7	6W5E_A	Beta-lactamase BSU-2	beta-lactamase activity, penicillin binding
		SLW subgroup (4)	
8	6N1N_A	Beta-lactamase STD-1	beta-lactamase activity, penicillin binding
		W-group (5)	
		SNW subgroup (4)	
9	2IWB_A	Methicillin resistance mecR1 protein	penicillin binding
		STW subgroup (1)	
10	1NRF_A	Regulatory protein BlaR1	penicillin binding
		G-group (23)	
		YNG subgroup (4)	

11	1YQS_A	D-alanyl-D-alanine carboxypeptidase	serine-type D-Ala-D-Ala carboxypeptidase activity
SNG subgroup (17)			
12	5ZQA_A	Lmo2812 protein	serine-type D-Ala-D-Ala carboxypeptidase activity
SCG subgroup (1)			
13	1ES5_A	DD-transpeptidase	serine-type D-Ala-D-Ala carboxypeptidase activity
YSG subgroup (1)			
14	1WYB_A	6-aminohexanoate-dimer hydrolase	6-aminohexanoate-dimer hydrolase activity
G-like group (39)			
YNY subgroup (Class C) (33)			
15	6FM6_A	Beta-lactamase TRU-1	beta-lactamase activity
YNA subgroup (1)			
16	1EI5_A	D-aminopeptidase	aminopeptidase activity
YLA subgroup (2)			
17	1CI9_A	Esterase EstB	hydrolase activity
YHQ subgroup (1)			
18	4IVK_A	Carboxylesterase	carboxylesterase activity
YPH subgroup (1)			
19	6KJC_A	Lovastatin esterase	hydrolase activity
SNM subgroup (1)			
20	2BG1_A	Penicillin-binding protein 1b	acyltransferase activity, penicillin binding, peptidoglycan glycosyltransferase activity, serine-type D-Ala-D-Ala carboxypeptidase activity
Q-like group (Class A) (6)			
SNQ subgroup (5)			
21	6V4W_A	Beta-lactamase CPA-1	N/A [3]
SNT subgroup (1)			
22	5TFQ_A	Beta-lactamase HGB-2	beta-lactamase activity
Inactive beta-lactamase group (2)			
GKN subgroup (2)			
23	5IHV_A	Beta-lactamase *B. ambifaria* MC40-6	beta-lactamase activity
Family: Glutaminase			
C-group (5)			
ONC subgroup (5)			

24	1U60_A	Glutaminase 1	glutaminase activity
Family: Dac-like			
G(Dac-like)-group (5)			
SNG subgroup (5)			
25	2EX2_A	D-alanyl-D-alanine carboxypeptidase DacB	carboxypeptidase activity, endopeptidase activity, penicillin binding, serine-type carboxypeptidase activity, serine-type D-Ala-D-Ala carboxypeptidase activity, serine-type endopeptidase activity

[1] Identification of the molecular function of the proteins were carried out using their PDB IDs, which contain the link to the UniProt server: https://www.uniprot.org/. UniProt Consortium. UniProt: the Universal Protein Knowledgebase in 2025. Nucleic Acids Res. 2025; 53(D1):D609-D617. doi: 10.1093/nar/gkae1010. [2] pH–isoelectric point; [3] N/A–Not Available.
